# Aluminum-Affecting Solidification Behavior, Microstructure, and Tensile Properties in Tin–Zinc Eutectic Alloy

**DOI:** 10.1021/acsomega.4c08359

**Published:** 2025-03-07

**Authors:** Ione Amorim Bezerra Neta, Bruno Silva Sobral, Raí Batista de Sousa, Jeverton Laureano Paixão, Suylan Lourdes de Araújo Dantas, José Eduardo Spinelli, Bismarck Luiz Silva

**Affiliations:** † Department of Materials Engineering, Federal University of Rio Grande do Norte, UFRN, Natal, RN 59078-970, Brazil; ‡ Department of Materials Engineering, Federal University of São Carlos, UFSCar, São Carlos, SP 13565-905, Brazil

## Abstract

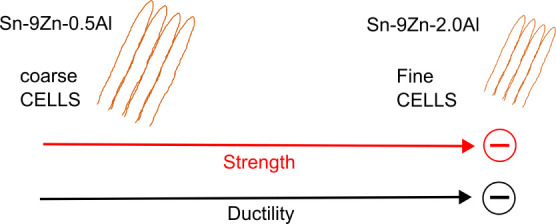

The Sn-9 wt %Zn eutectic alloy is a promising material as a soldering alloy. It has a eutectic temperature (198 °C) close to the Sn–Pb eutectic alloy, low toxicity, low cost, and excellent mechanical properties. However, the Sn–Zn alloys exhibit disadvantages such as low corrosion resistance and low wettability, which limits their practical use in soldering operations. One of the improvement alternatives is modification with aluminum (Al). This element promotes changes in the microstructure, mechanical properties, and corrosion resistance. Therefore, the present work aims to study the effect of adding Al (0.5% and 2.0% in wt %) on the solidification thermal parameters (cooling rate-Ṫ_L_ and growth rate-V_L_), microstructure, macrosegregation, tensile properties, and fracture modes of directionally solidified (DS) Sn-9 wt % Zn alloys. The samples have been characterized by optical microscopy (OM), scanning electron microscopy (SEM), X-ray fluorescence (XRF), X-ray diffraction (XRD), and tensile tests. Al additions to the Sn-9 wt % Zn alloy increase the *liquidus* (T_L_) and reduce the eutectic (T_E_) temperatures, thus increasing the solidification interval. Eutectic cell growth has been observed for the Sn–Zn–Al alloys, with cells composed of β-Sn, α-Zn and α-Al phases. Only the Sn-9 wt % Zn-2 wt % Al alloy promotes a change in the microstructural scale, significantly refining the eutectic cellular arrangement. The morphologies of the α-Zn phase particles forming the eutectic constituent are shown to be dependent on the Al content as well as on the cooling rate. The Portevin-Le Chatelier (PLC) effect and the twinning deformation mechanism have been observed in the stress–strain curve features in Sn–Zn–Al alloys, being impacted by the Al content. The Al additions promote a reduction in both the ultimate tensile strength (σ_u_) and the elongation-to-fracture (δ). In comparison to the Sn-9 wt % Zn binary alloy, Al has not changed the fracture mode, remaining ductile, in the Sn–Zn–Al alloys as well. The major contribution lies in the wide variety of samples solidified at significantly different cooling rates, along with the two additions of Al for mapping, which corroborates industrial scales of soldering conditions.

## 1 Introduction

The Sn–Pb soldering alloys have shown great potential for use at various levels of industrial assembly due to their excellent mechanical properties and low cost.^1,2^ However, several countries and economic blocs have adopted guidelines that prevent the use of alloys containing lead.^3,4^ The toxicity of this metal have represented a danger to the environment and human health.^5,6^ Therefore, the development of lead-free soldering alloys that meet industry needs has become a global concern.

In recent years, most research has been dedicated to the development of different soldering alloys such as Sn–Zn, Sn–Bi, Sn–Cu, Sn–Ag, Sn–In, among others. These systems have emerged as potential replacements for Sn–Pb system alloys.^7,8^ In this context, the eutectic Sn-9 wt % Zn alloy has attracted attention for applications in electronic microcomponents due to its inherent characteristics, such as melting temperature (198.00 °C) close to the Sn-38 wt % Pb alloy, low cost, and suitable mechanical properties.^9−12^ The microstructure of the Sn-9 wt % Zn alloy consists of eutectic colonies formed by zinc-rich and tin-rich phases. The zinc phase can appear in two morphologies, depending on the experienced cooling rate during solidification: globular and needlelike.^13^ However, low corrosion resistance and low wettability restrict a more effective use of these Sn–Zn alloys.^9^

In order to overcome such disadvantages of Sn–Zn alloys, researchers added a third element, such as Ga, Bi, Al, and Ag.^1,2^ Al is of particular interest due to its remarkable ability to refine grains and improve mechanical strength. Studies showed that the addition of Al to Sn–Zn alloys improved corrosion resistance due to the formation of an Al_2_O_3_ film.^11,12,14,15^ Lin et al.^16^ and Zhang et al.^17^ reported that the addition of Al in the Sn-9 wt % Zn alloy improved the wettability level on metallic substrates. Significant improvements in tensile strength and hardness were also demonstrated with additions of Al in Sn–Zn based alloys.^8,18^ El Basaty et al.^19^ reported a microstructure consisting of the β-Sn, α-Zn (in needle morphology) and Al_6_Zn_3_Sn phases for the Sn-9 wt % Zn-0.5 wt % Al alloy. Das and coauthors^18^ described a similar microstructural arrangement for the same chemical composition, highlighting that the addition of Al (0.5 in wt %) reduced the size of the α-Zn phase. Furthermore, it was observed that the Al_6_Zn_3_Sn intermetallic compound was uniformly distributed in the Sn–Zn eutectic. Recently, Xinhan et al.^20^ reported Sn–Zn–Al alloys as potential materials for joining between Sn-based alloys and ceramics such as alumina (Al_2_O_3_).

The majority of the solder alloy properties are determined by the state of the microstructure, therefore, a fundamental understanding of the microstructure evolution along the solidification of solder alloys is of critical importance.^21−25^ This is also critical for Sn–Zn alloys.

In accordance with the above and considering that there is a gap in studies that correlate the solidification behavior in a variety of conditions, microstructure, mechanical properties and fracture modes of Sn–Zn–Al alloys applied in soldering operations, the present work aims to investigate and understand the effect of Al additions (0.5% and 2.0% wt %) on the directionally solidified (DS) Sn-9 wt % Zn eutectic alloy. Experimental relationships such as microstructure-cooling rate and microstructure-mechanical properties will be discussed, in addition to fracture mechanisms in tensile loading mode, considering the tested Al contents.

## 2 Experimental Procedure

### 2.1 Solidification Methods and Thermodynamic Calculations

In order to prepare the alloys, commercially pure metals such as tin (99.95 wt %), zinc (99.94 wt %) and aluminum (99.795 wt %) were cut into small pieces using a Franho band saw, model FM. 18-S. The properly cut metals were weighed with the aid of a Shimadzu digital scale, model BL3200H, with an accuracy of 0.01 g, in order to produce Sn–Zn–Al alloys with nominal chemical compositions: Sn-9 wt % Zn-0.5 wt %Al and Sn-9 wt % Zn-2.0 wt % Al.

In the melting stage, the metals (Sn, Zn, Al) were inserted into a silicon carbide crucible (2.0 L) internally coated with an alumina-based suspension layer, using the Carborundum specification model QF-180, so that contamination of the alloys could be avoided and the durability of the container increased. The crucible with the metals was inserted into a YUELON induction furnace, model MF-35. The liquid metal was homogenized and then poured into two portions.

First, a small volume was poured into a silicon carbide crucible (300 mL) wrapped in a refractory glass fiber blanket, aiming to acquire a slow cooling curve to determine main phase transformation temperatures. A type J thermocouple connected to the Keysight brand and Agilent 34901 data acquisition system, with the resolution of two temperature readings per second coupled to a computer, it was inserted into the crucible, recording the temperature variation over time;

Second, the remaining liquid metal (largest volume) was poured into an ingot-mold and bottom-plate assembly, considering a superheat of 10% above the *liquidus* temperature (T_L_). The ingot-mold is split and made of AISI 310 stainless steel with an internal diameter of 60 mm, a height of 160 mm and a wall thickness of 7 mm. It has nine lateral holes of 1.6 mm in diameter for the passage of the type J thermocouples. The internal walls of the ingot-mold were internally coated with an alumina-based suspension layer, following the Carborundum model QF-180 specifications to avoid thermal exchanges, to facilitate the demolding of the casting and to avoid contamination of the alloy. The bottom-plate is made of SAE 1020 low carbon steel, having 100 mm in diameter and 3 mm thick. A surface finish was carried out with sequential sanding from 150 to 2000 mesh.

**Figure 1:**
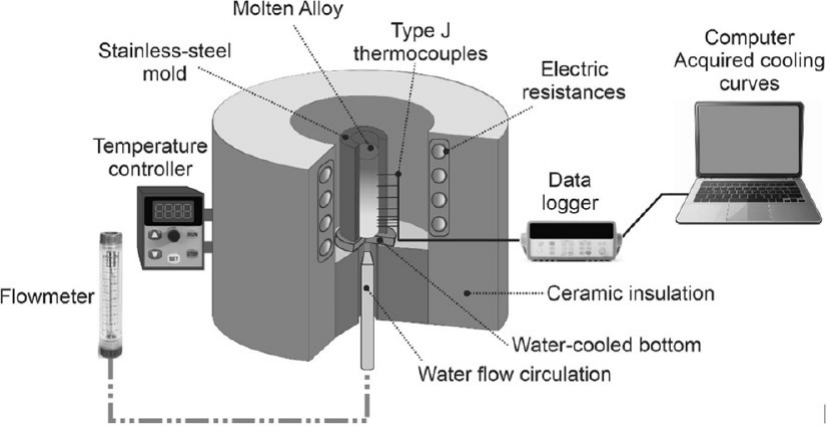
Schematic representation of the vertical upward unidirectional solidification device.

The Sn–Zn–Al castings were obtained using a vertical upward unidirectional solidification device whose details can be found elsewhere,^26−28^ as shown in Figure 1. Thermocouples have been inserted along the mold at positions 5 mm, 10 mm, 15 mm, 20 mm, 25 mm, 45 mm, 55 mm, 70 mm and 90 mm from the metal/mold interface.

Both the growth rate of the *liquidus* isotherm (V_L_) and the cooling rate (Ṫ) at each position of each thermocouple were determined using procedures previously published.^29,30^

Using the CALPHAD (CALculation of PHAse Diagrams) based on the TCSLD3 database, thermodynamic calculations were performed to obtain information about the solidification path, phase stoichiometries, transformation temperatures and the solubility limits in the Sn-9Zn–Al alloys. The generation of the isopleth for the Sn–Zn–Al system considered a percentage of Al varying from 0% to 2.5% (in wt %), fixing the Zn content at 9% (in wt %). Furthermore, solidification paths were simulated using the Scheil model for nonequilibrium processes.

### 2.2 Microstructural Characterization

In the microstructural analysis, transverse and longitudinal cuts were made using a saw blade at positions 5 mm, 10 mm, 15 mm, 20 mm, 25 mm, 30 mm, 40 mm, 50 mm, 60 mm, 70 mm and 90 mm from the metal/mold interface. The samples were cold embedded in polyester resin, manually sanded from 150 to 1200 mesh sandpaper and subsequently polished with 1 μm diamond paste using a rotary polisher (Teclago PVVD). The microetching was carried out by immersion in a solution of 92 m*l* CH_3_OH, 5 m*l* HNO_3_ and 3 m*l* HCl for a period of 1 min. The micrographs were recorded using a Nikon Eclipse MA2000 optical microscope.

Measurements of the eutectic cell (λ_C_) and interphase (Zn needles-λ_n_) spacings were carried out using the method proposed by McCartney and Hunt,^31^ according to the equation , where “λ” represents the microstructural spacing, “L” represents the predefined length of the reference line and “n” the number of eutectic cells or intersections in the α-Zn phase along the distance L.

**Figure 2:**
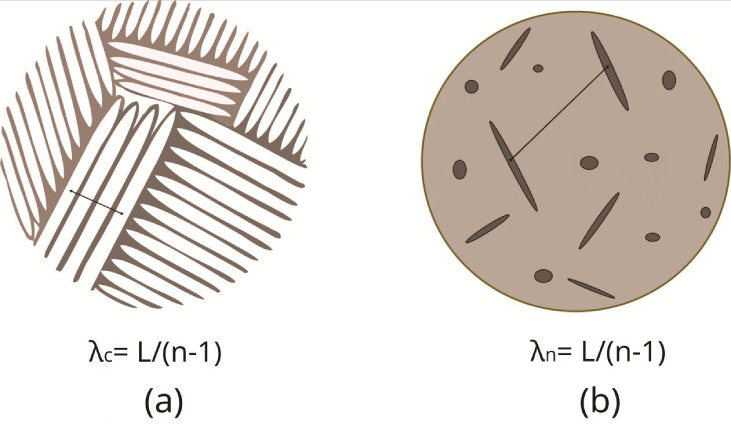
Schematic representation of the linear intercept method used to quantify the (a) eutectic cell (λ_C_) and (b) interphase (λ_n_) spacings in Sn–Zn–Al castings.

Measurements of λ_C_ and λ_n_ have been carried out on micrographs acquired by optical microscopy (OM) and scanning electron microscopy (SEM), respectively. A minimum of 40 measurements for each selected position have been carried out, obtaining mean and standard deviations. Figure 2 shows a representation of the method used in the measurements.

The microstructures of the studied alloys were investigated by scanning electron microscopy (SEM), in order to characterize the constituent phases of the eutectic mixtures regarding to their size, morphology and distribution. Local chemical analysis and chemical mapping were carried out using the energy dispersion spectroscopy (EDS) technique coupled to SEM, in order to qualitatively identify the chemical elements and the positioning of these elements in the microstructures throughout each ingot.

In order to reveal the microstructure, the microetching was carried out for a longer period of 2 min in the transversal samples. The images were obtained using a Carl Zeiss model Auriga 40 electron microscope, employing a secondary electron detector mode. SEM analyzes have also been used to acquire the fracture surfaces of the specimens after the tensile test, aiming to verify the effects of the microstructure and element contents (Zn, Al and Bi) on the fracture mechanisms.

X-ray fluorescence (XRF) analyzes were carried out with the aim of identifying the variation in zinc (Zn) and Al contents along the length of the Sn–Zn–Al castings. The equipment used was Shimadzu, EDX-720 model. X-ray diffraction (XRD) analyzes were carried out to identify the phases in the as-cast microstructures along the Sn–Zn–Al castings. The analyzes were carried in four samples of each casting encompassing different levels of cooling rates. The tests were carried out using an X-ray diffractometer from Shimadzu, model XDR-700. Cu–Kα radiation was used at a wavelength (λ) of 0.15406 nm, with a scan of 20–90°, and a scan speed of 1°/min.

### 2.3 Tensile Tests

The tensile tests were carried out with the purpose of determining the tensile properties as a function of the microstructural features and parameters throughout each casting, in order to establish experimental correlations that describe each mechanical behavior. The specimens used in the tensile tests were prepared and tested, according to the specifications of the ASTM/E8–8 M standard,^32^ at room temperature (24 °C) with a strain rate of 1.67 × 10^4^ s^–1^, using a universal machine Shimadzu, AG-X 300 kN model, operated with the Trapezium X supervisory software.

**Figure 3:**
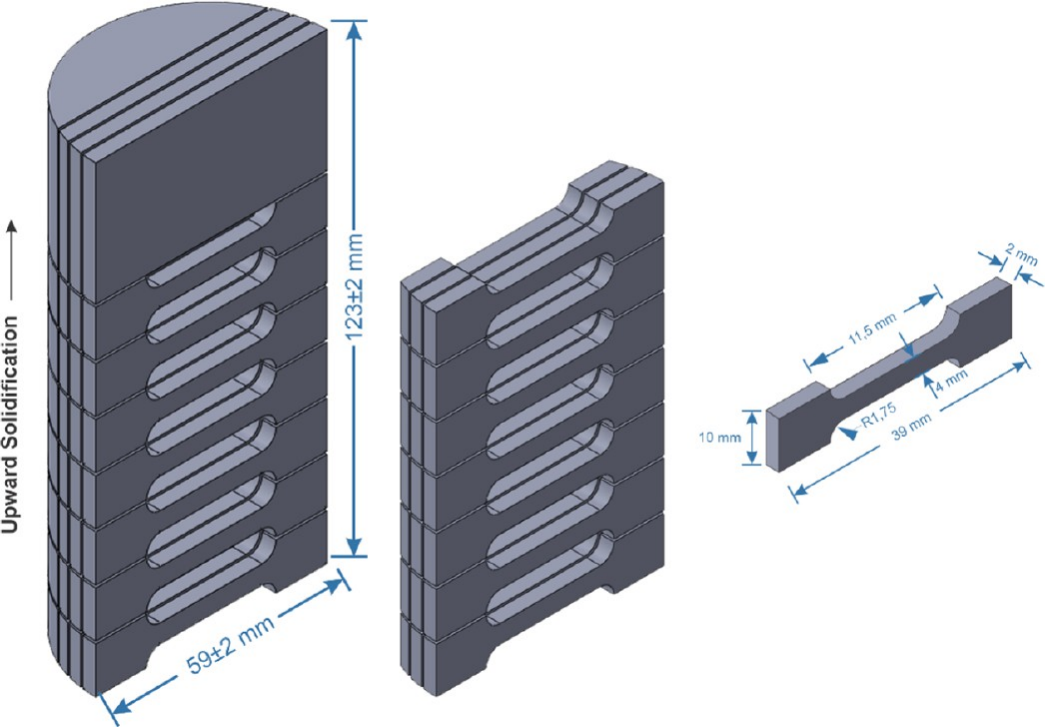
Test specimen extraction across de DS casting scheme for tensile tests.

Specimens were extracted from the directionally solidified castings, at different positions along their lengths, as shown in Figure 3. The positions for removing specimens from the metal/mold interface were: 0–12 mm (6 mm), 14–26 mm (20 mm), 28–40 mm (34 mm), 42–54 mm (48 mm), 56–68 mm (62 mm), 70–82 mm (76 mm), 84–96 mm (90 mm). For each position, three (3) specimens were obtained with the aim of guaranteeing the reproducibility of the results, and extracting mean and standard deviation values.

## 3 Results and Discussion

### 3.1 Thermal Profiles, Process Parameters, and Solidification Paths

**Figure 4:**
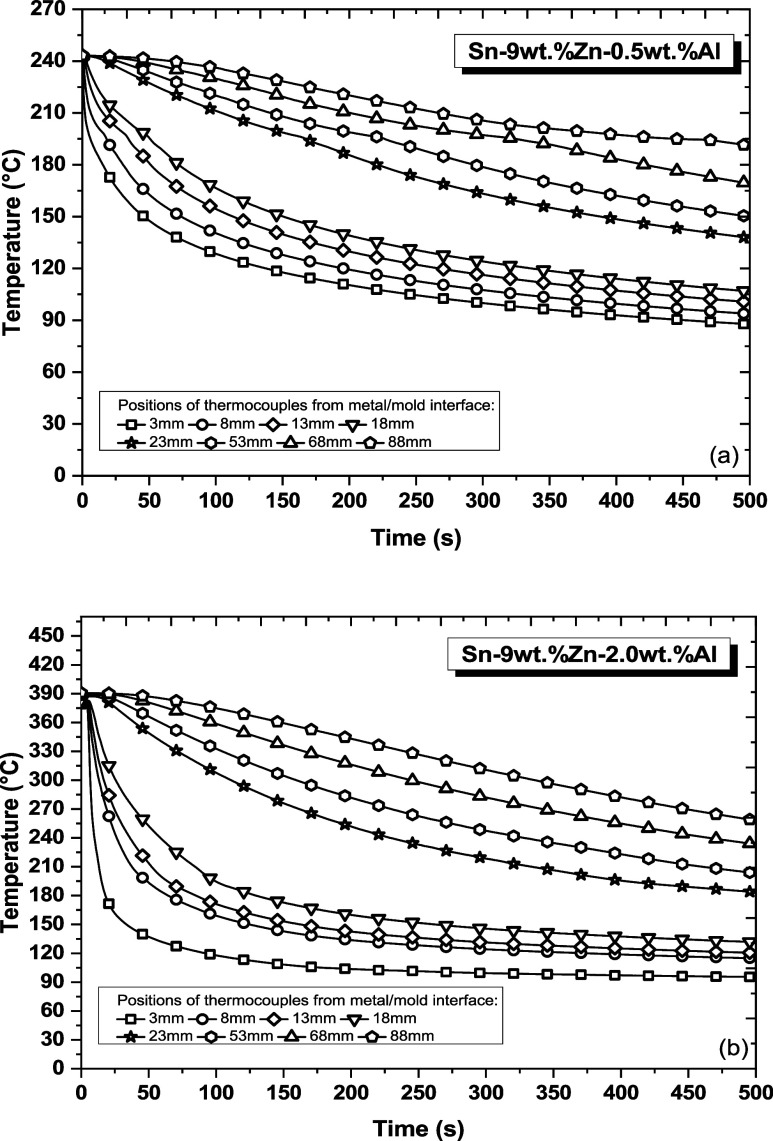
Experimental thermal profiles measured in the (a) Sn-9 wt %Zn-0.5 wt %Al and (b) Sn-9 wt %Zn-2.0 wt %Al alloys.

The thermal profiles recorded during the directional solidification of Sn–Zn–Al alloys are presented in Figure 4. Each curve represents a thermocouple along the length of each alloy casting. It can be verified that the cooling curves present higher temperature variations with time when associated with the positions closest to the metal/mold interface, indicating different ranges of cooling rates throughout the studied alloys castings, which will reflect on microstructural length-scales, distribution and morphology of phases. This data on temperature variation over time were used to calculate solidification thermal parameters such as growth rate of the *liquidus* isotherm (V_L_) and cooling rate (Ṫ_L_).^29,30^

**Figure 5:**
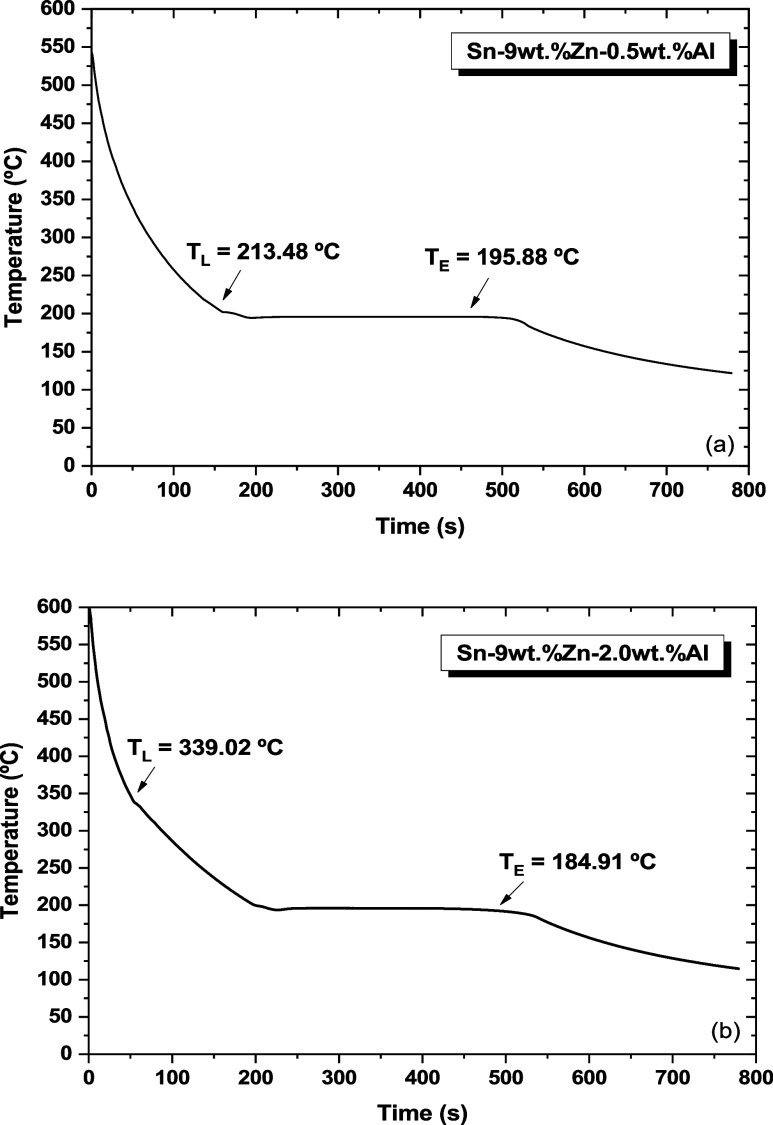
Experimental cooling curves for the (a) Sn-9 wt %Zn-0.5 wt %Al and (b) Sn-9 wt %Zn-2.0 wt %Al alloys.

Figure 5 presents the experimental cooling curves for Sn–Zn–Al alloys. Through the curves it was possible to identify the values of the *liquidus* (T_L_) and eutectic (T_E_) temperatures, respectively. It is observed that the phase transformation temperatures are similar to the theoretical values obtained by thermodynamic simulation via the Thermo-Calc software. This indicates compositional levels close to nominal values and also the authenticity of the database in the calculations.

It is noted that the increase in Al content promoted an increase in T_L_ from 213.48 to 339.02 °C and a reduction in T_E_ from 195.88 to 184.91 °C. Compared to the binary Sn-9 wt %Zn alloy studied by Ramos et al.,^13^ it is observed that the addition of Al can change the solidification interval.

**Figure 6:**
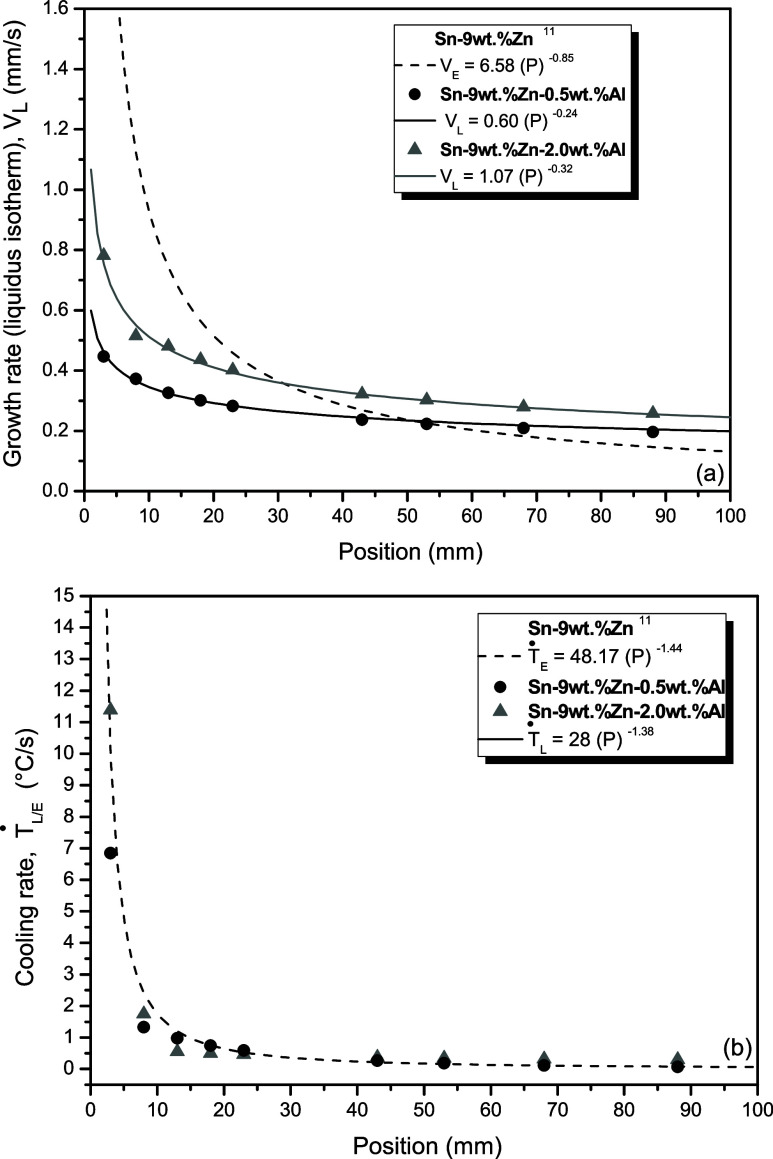
Evolutions of the (a) growth rate (V) and (b) cooling rate (Ṫ) as a function of position (P) from the metal/mold interface for the Sn–Zn–Al alloys.

The variations of growth rate (V_E_/_L_) and cooling rate (Ṫ_E_/_L_) with the position considering the *liquidus* isotherm and the eutectic front are presented in Figure 6. The data of the DS Sn-9 wt % Zn alloy under nonequilibrium reported by Ramos et al.^13^ were used for comparison purposes. It is observed that V_L_ and Ṫ_L_ decreased as the *liquidus* isotherms of the analyzed alloys move away from the metal/mold interface. This is attributed to the progressive growth of the solidified layer and consequently, the increase in thermal resistance incorporated throughout the solidification process,^29,30,33^

The purely eutectic coupling of the binary alloy resulted in higher growth rates compared to the displacement of primary phase isotherm in the ternary alloys. This is because the eutectic diffusive coupling can be highly efficient, accelerating the growth of its constituent phases.

Compared to the Sn-9 wt % Zn alloy,^13^ alloys with Al additions revealed a decrease in V_L_, especially in the initial positions (up to 30 mm), where the greatest variations in thermal parameters occur due to lower thermal resistance introduced by the formation of successive solid layers. From 50 mm onward from the metal/mold interface, similar values are observed, where heat extraction is already very low. This indicates that the solute content can be a factor in reducing the solidification rate with the position considering the *liquidus* isotherm. According to Kurz and Fisher,^33^ this decrease in V at the beginning of the ingot may be related to the insertion of a solute that slows down the growth rate at the solid–liquid interface.

Among the Sn–Zn–Al alloys, it is noted that the increase in Al content caused an increase in the cooling rate in the position closest to the metal/mold interface. This can be associated with a higher heat extraction efficiency in the metal/mold system and, consequently, an increase in the interfacial heat transfer coefficient.^34^

**Figure 7:**
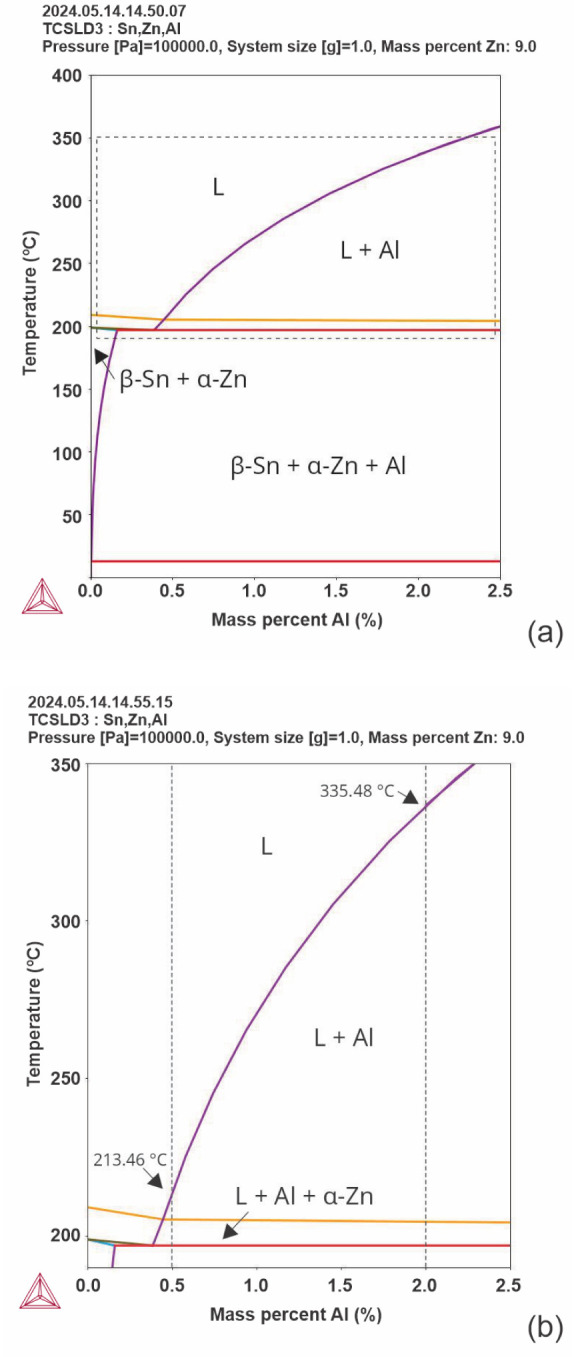
(a) Isopleth of the Sn-9 wt %Zn-xAl system calculated by the Thermo-Calc software (TCSLD3 database). The vertical dashed lines (black) indicate the alloys examined in the present study, and (b) enlargement of the highlighted area in (a).

The result of the isopleth calculation for the Sn–Zn–Al system can be seen in Figure 7. In Figure 7a, the expanded area in Figure 6b is highlighted (by a rectangle with black dashed lines). It is observed that the increase in Al content from 0.5% to 2.0% caused an increase in the *liquidus* (T_L_) temperature from 213.46 to 335.48 °C, while the *solidus* temperature (T_s_) remained at 197.01 °C. According to Smetana et al.,^35^ the increase in Al content in the Sn-6.7%Zn-0.5%Al alloy to Sn-6.7%Zn-2.5%Al (in wt %) promoted an increase in T_L_ from 296.3 to 379.40 °C, which is in agreement with the present observations.

According to Lu et al.,^36^ with increasing solidification interval and solute content, the degree of segregation becomes severe. Flemings^37^ reported that reverse segregation can occur due to solidification contraction, and is favored by slow cooling rates, longer solidification intervals, and coarse dendritic spacings. Hulka et al.^38^ reported that the interdendritic segregation is similarly reduced by reducing the solidification interval.

**Figure 8:**
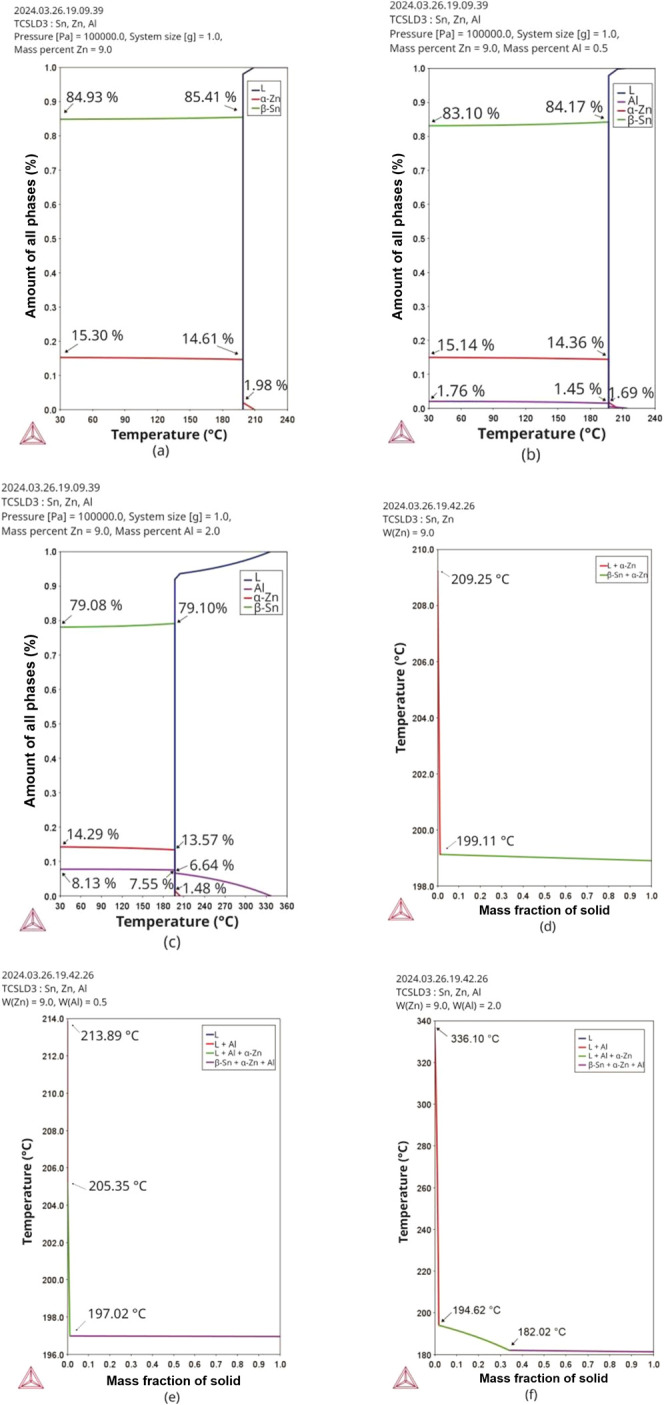
Variation in the mass fraction of the phases in the (a,d) Sn-9 wt %Zn, (b,e) Sn-9 wt %Zn-0.5 wt %Al and (c,f) Sn-9 wt %Zn-2.0 wt %Al alloys as a function of temperature, calculated using the Thermo-Calc software (TCSLD3 database) in equilibrium (a,b,c) and nonequilibrium thermodynamics (d,e,f).

The computation of the mass fraction of the phases was carried out in order to investigate the change of each phase throughout the solidification process. Figure 8 shows such evolutions for the Sn–Zn and Sn–Zn–Al alloys considering thermodynamic equilibrium and nonequilibrium conditions (Scheil model). In the Sn–Zn alloy (Figure 8a), it is noted that the nucleation of the Zn-rich (α-Zn) phase occurs at 209 °C, quickly followed by the formation of the eutectic Sn + Zn at a temperature of 199.5 °C. These temperatures are similar to those demonstrated using the Scheil model (Figure 8d). This nonequilibrium solidification model represents better the soldering process, as it assumes infinitely fast diffusion in the liquid phase and zero or limited diffusion within the solid phases.^39,40^

The final mass fractions of the phases rich in Zn and Sn were 15.3% and 84.93% for the binary alloy, respectively. Based on the solidification paths provided by the Scheil model presented in Figure 8a, it is observed that for the Sn-9 wt %Zn alloy, the first phase formed is rich in zinc (with a compact hexagonal structure), followed by the eutectic formed by Zn and Sn (with a body-centered tetragonal structure).^41^

Considering the modified Sn-9 wt %Zn-0.5 wt %Al alloy, it is observed that the Al-rich phase (lilac line) is the first to be formed at 213.07 °C, followed by the Zn-rich phase (red line) at 205.12 °C. Soon after, the ternary eutectic Sn+Zn+Al forms at 197.14 °C. The addition of 0.5 wt %Al to the Sn-9 wt %Zn alloy did not promote significant changes in the amounts of the final Zn-rich and Sn-rich phases as compared to the binary alloy. The increase in the Al content from 0.5% to 2.0% (in wt %) caused an increase in the nucleation temperature of the Al-rich phase, from 213.07 to 338.15 °C, however, following the same phase formation dynamics as demonstrated for the Sn-9 wt %Zn-0.5 wt %Al alloy. This increase in Al content slightly decreased the solid fractions of the Sn-rich and Zn-rich phases at 30 °C from 83.10% to 79.08%, and from 15.14% to 14.29%, respectively.

### 3.2 Microstructures and Cellular/Eutectic Growth Laws

**Figure 9:**
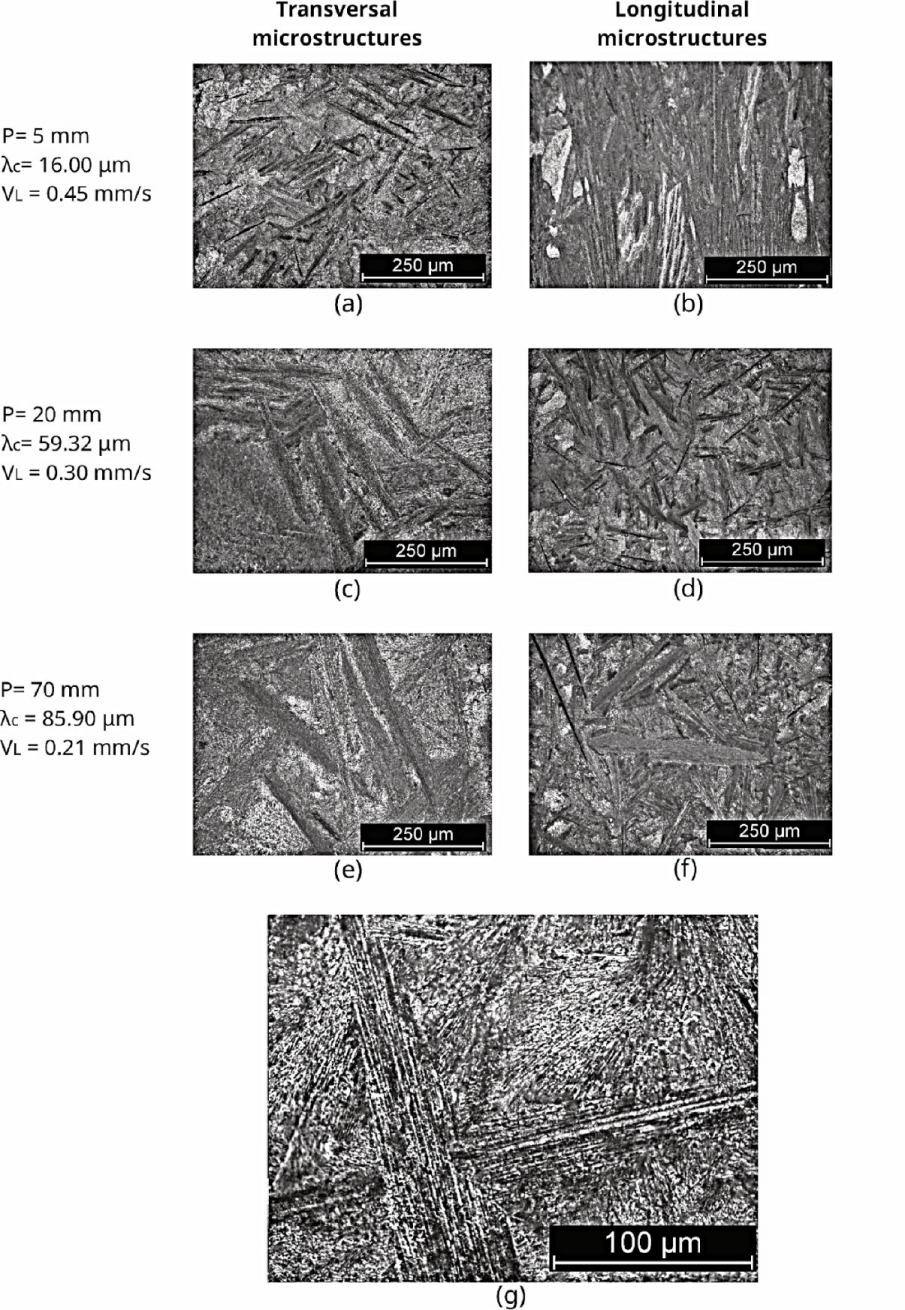
As-cast microstructures (transversal–left (a,c,e) and longitudinal–right (b,d,f)) for the Sn-9 wt %Zn-0.5 wt %Al alloy, showing the cell spacing (λ_c_) and thermal parameter (V_L_), where P is the position from the metal/mold interface. (g) is a high-magnified optical image.

**Figure 10:**
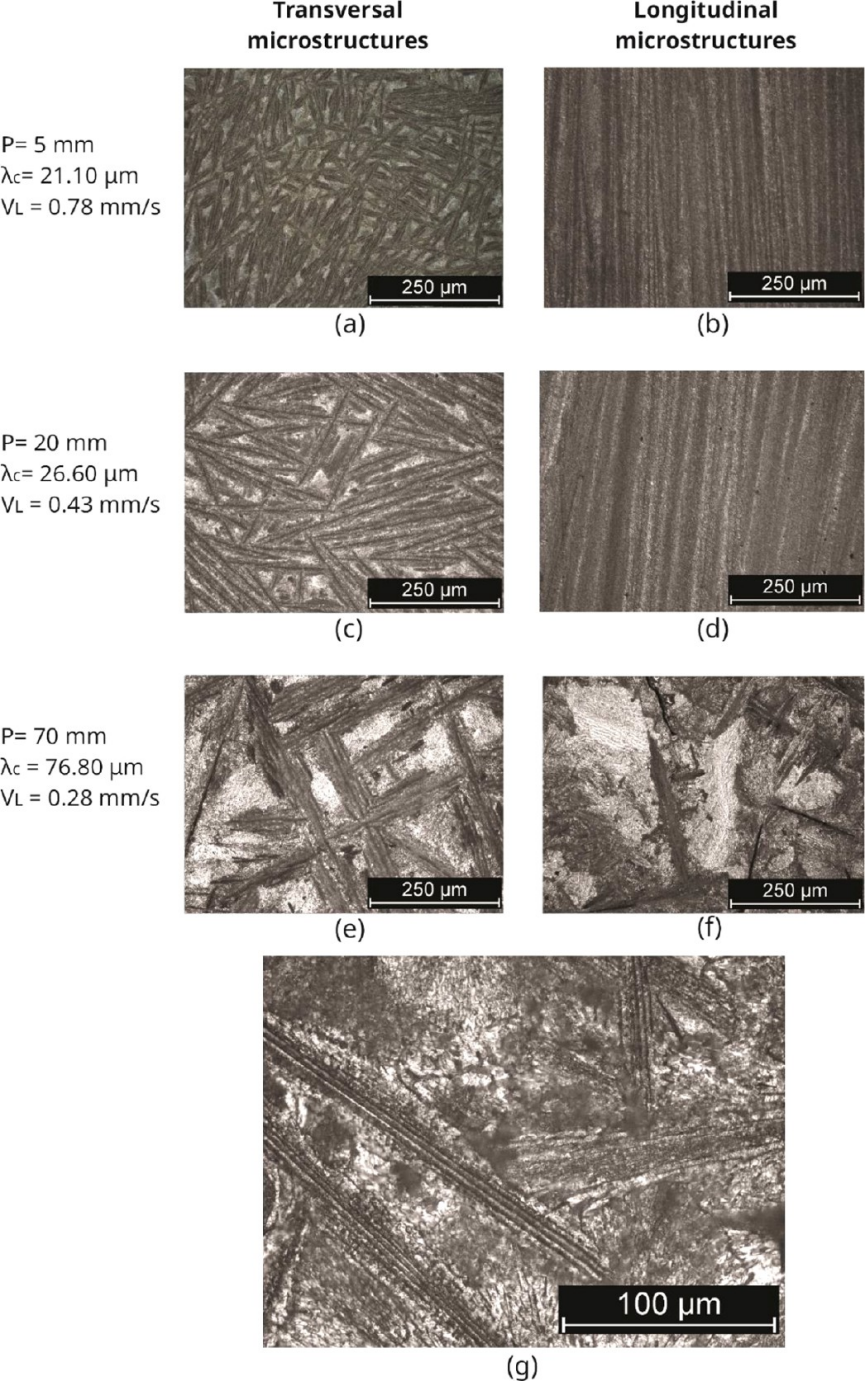
As-cast microstructures (transversal–left (a,c,e) and longitudinal–right (b,d,f)) for the Sn-9 wt %Zn-2.0 wt %Al alloy, showing the cell spacing (λ_c_) and thermal parameter (V_L_), where P is the position from the metal/mold interface. (g) is a high-magnified optical image.

Figures 9 and 10 display the as-cast microstructures obtained by optical microscopy from the analysis of transversal and longitudinal sections, also mentioning the thermal solidification parameters (growth rate-V) and associated microstructural (eutectic cell spacing-λ_C_). It was noted that both Sn–Zn–Al alloys presented eutectic cells formed by the β-Sn, α-Zn and α-Al phases. These microstructures feature elongated cells of the Zn-rich phase (α-Zn) in the form of plates with a eutectic mixture. The α-Zn-rich phase and the eutectic appear to grow in a coupled manner. According to Kurz and Fisher,^33^ cellular morphology promotes greater compositional homogeneity, better phase distribution, lower level of segregation and higher levels of mechanical resistance compared to the dendritic arrangement.

**Figure 11:**
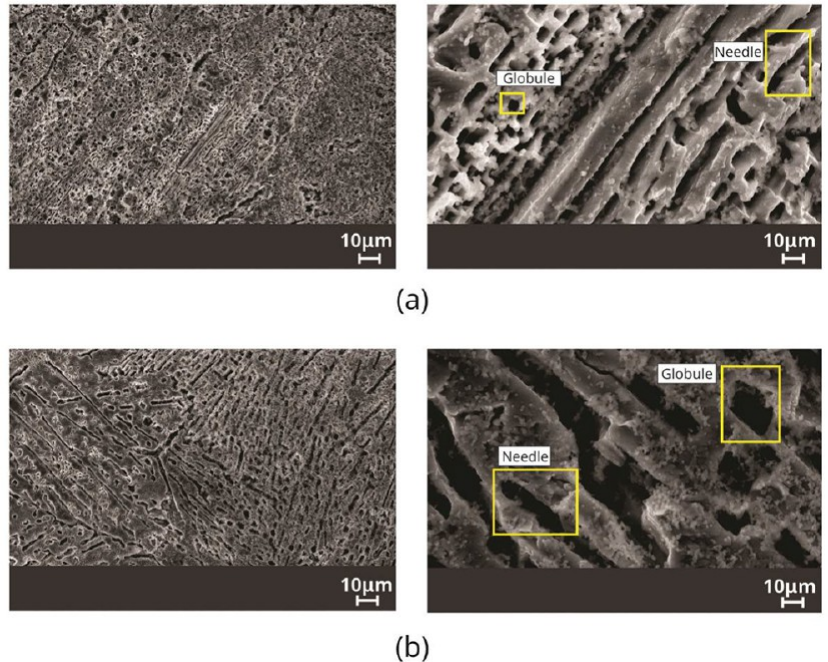
SEM images in the Sn-9 wt %Zn-0.5 wt %Al alloy, highlighting the morphologies of the α-Zn phase, considering the positions (a) 5 mm and (b) 70 mm from the metal/mold interface.

**Figure 12:**
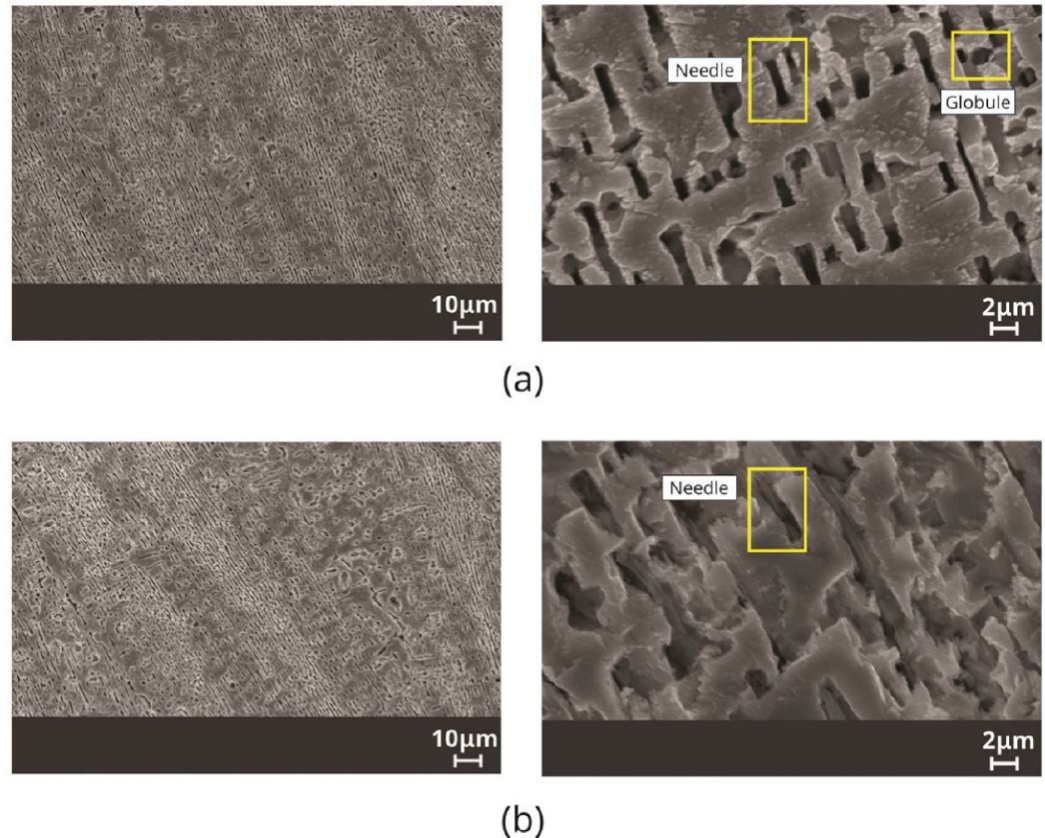
SEM images in the Sn-9 wt %Zn-2.0 wt %Al alloy, highlighting the morphologies of the α-Zn phase, considering the positions (a) 5 mm and (b) 70 mm from the metal/mold interface.

Figures 11 and 12 show images obtained by scanning electron microscopy (SEM) from transverse positions 5 mm and 70 mm from the metal/mold interface for the Sn–Zn–Al alloys. The results of the nonequilibrium solidified Sn-9 wt %Zn alloy reported by Ramos et al.^13^ have been considered to discuss the effects of Al in the Sn–Zn alloys.

In the Sn-9 wt %Zn-0.5 wt %Al alloy, it is noted that the phase rich in Zn located inside eutectic cells, α-Zn, shows two types of morphology, globules and needles. Both morphologies predominated at positions closest to the metal/mold interface, 5 mm and 20 mm, for V_L_ > 0.30 mm/s values. On the other hand, Zn needles prevail for the most distant positions closest to the metal/mold interface, 70 mm, for V_L_ < 0.21 mm/s. Considering the Sn-9 wt %Zn-2.0 wt %Al alloy, it is observed that the α-Zn phase also has two morphologies, globules and needles. Both morphologies predominate at positions closest to the metal/mold interface, 5 mm and 20 mm, for V_L_ > 0.43 mm/s. Meanwhile, Zn needles dominated for the furthest positions, 70 mm, corresponding to V_L_ < 0.28 mm/s.

Garcia and collaborators^42^ reported that a needlelike morphology predominated for V_E_ < 0.45 mm/s in a directionally solidified (DS) Sn-9 wt %Zn eutectic alloy. Similarly, Ramos et al.^13^ also reported that for a Sn-9 wt %Zn alloy, the needlelike morphology predominated for positions furthest from the metal/mold interface and for V_E_ < 0.45 mm/s. Moreover, globules and needles were reported to predominate in the closest positions to the metal/mold interface for V_E_ > 0.45 mm/s.

According to Song et al.,^43^ the alloying element can dissolve in the eutectic structure and cause changes in the lattice parameters of Sn and Zn, and consequently induce an increase in entropy of the solution, which can result in the morphological change of the Sn–Zn eutectic, changing from α-Zn needle particles to coarse flakes.

**Figure 13:**
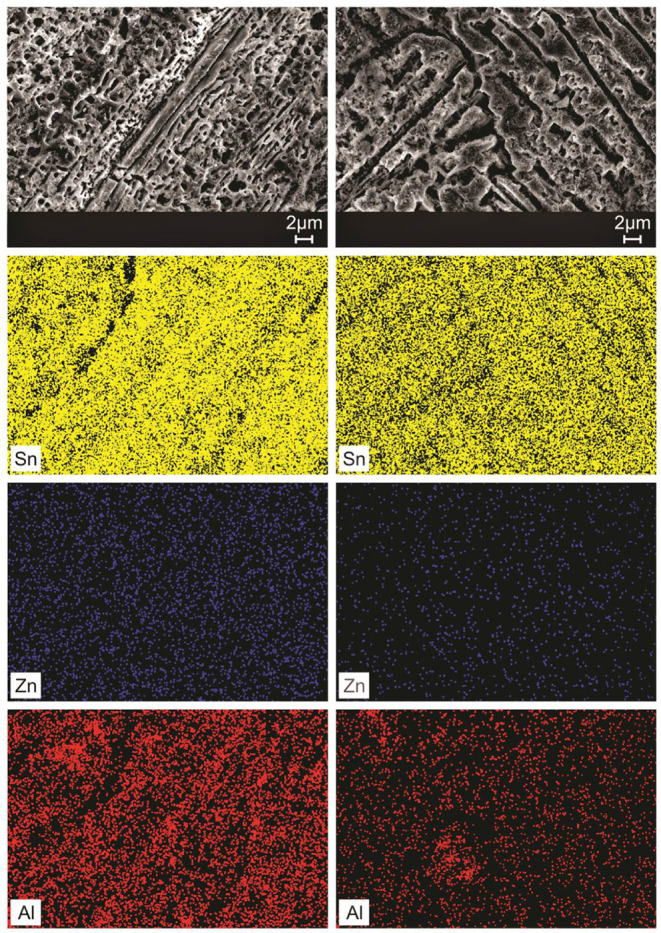
SEM/EDS elemental maps along transverse samples for positions 5 mm and 90 mm from the metal/mold interface in the Sn-9 wt %Zn-0.5 wt %Al alloy.

**Figure 14:**
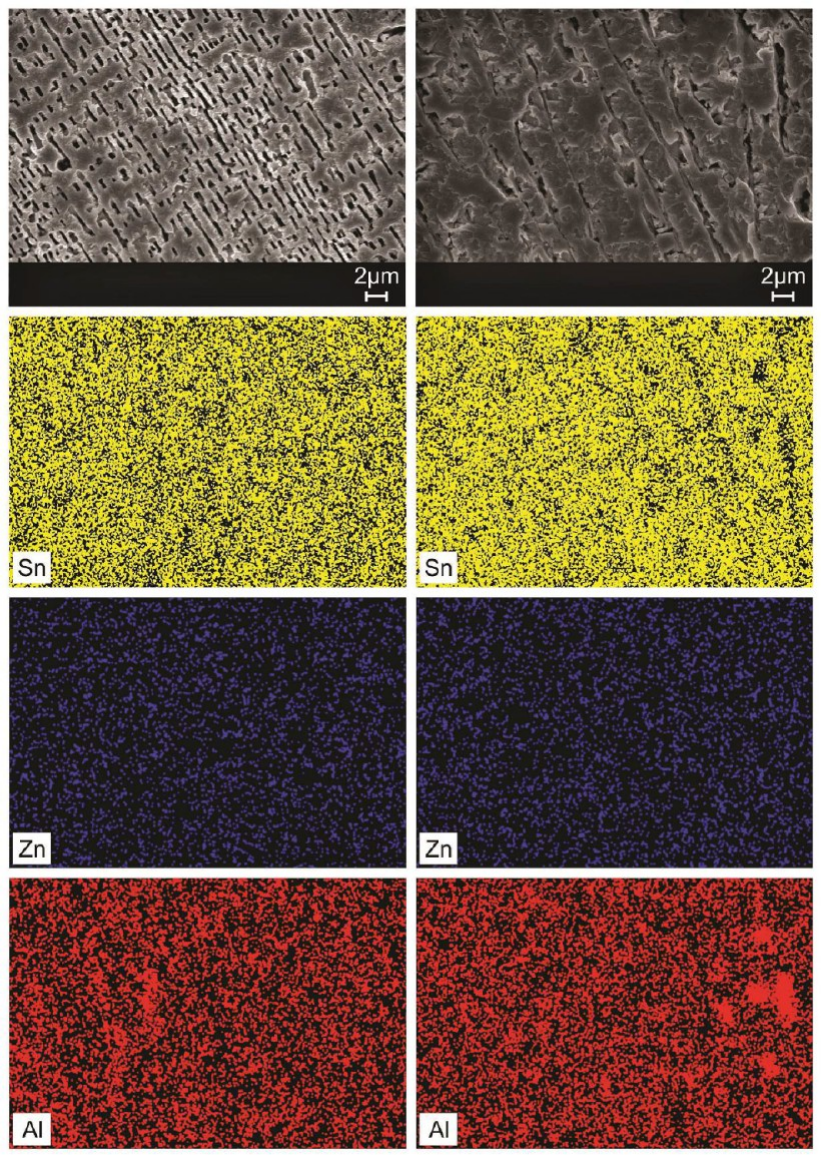
SEM/EDS elemental maps along transverse samples for positions 5 mm and 90 mm from the metal/mold interface in the Sn-9 wt %Zn-2.0 wt %Al alloy.

The elementary chemical mappings in the as-cast microstructures of the Sn–Zn–Al alloys were carried out via SEM/EDS, in a qualitative way, as shown in Figures 13 and 14.

In the microstructures of Sn–Zn–Al alloys, the presence of Sn (in the yellow-colored region), Zn (in the blue-colored region) and Al (in the red-colored region) can be noted. The Sn and Al contents are preferentially in the eutectic region, as expected. The Zn was consumed both in the formation of needles and globules (darker regions of the grayscale SEM image) and in the eutectic constituent. This may be associated with the low solid solubility of Zn in Sn, around 0.6% (by weight).^44^ Das et al.^18^ reported the effects of adding 0.5% Al (in wt %) on the microstructure of the Sn-9 wt %Zn alloy and observed that the Sn and Al atoms were concentrated in the eutectic regions. Sobral et al.^30^ demonstrated for the Sn-3.5 wt %Ag-0.5 wt %Zn and Sn-3.5 wt %Ag-1.0 wt %Zn alloys that Sn atoms are arranged inside the eutectic cells.

**Figure 15:**
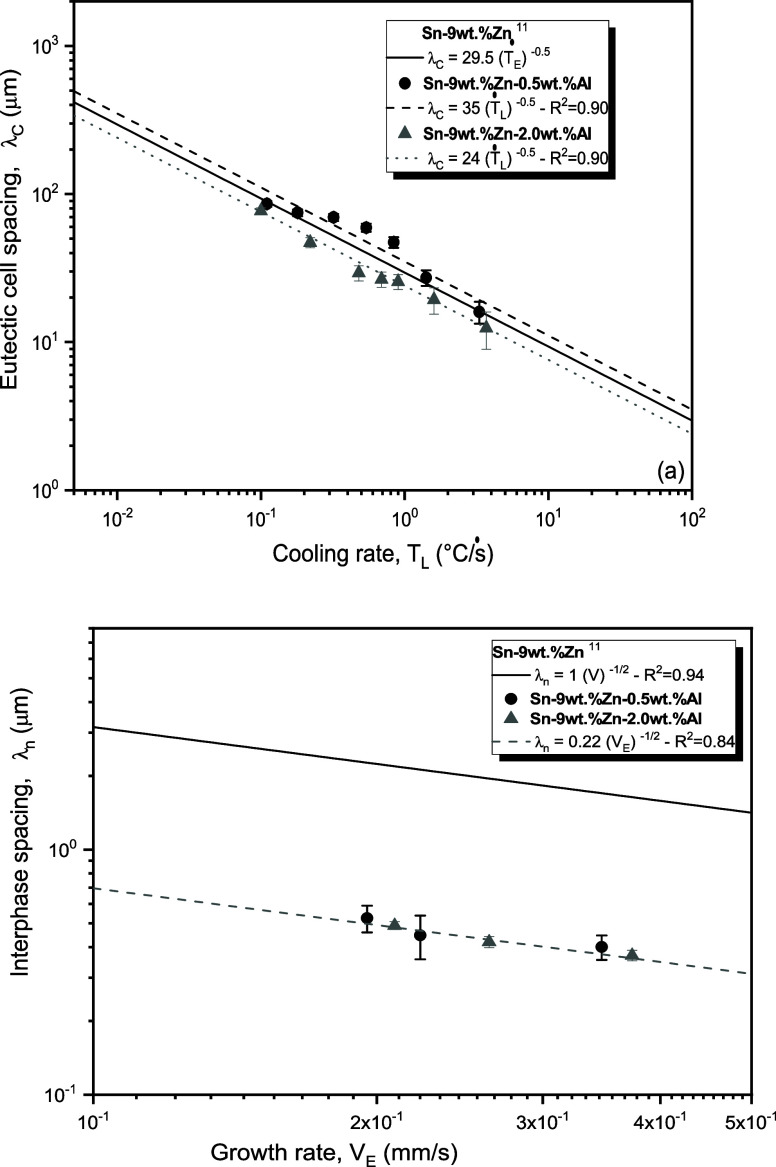
Evolution of (a) eutectic cell spacings (λc) as a function of cooling rate (TL), and evolution of (b) Zn needle spacings (λ_n_) as a function of growth rate (V_E_) for the Sn–Zn–Al alloys.

Figure 15 shows the evolutions of the eutectic cell spacings (λ_c_) and interphase spacings (λ_n_: α-Zn phase in needlelike morphology) as a function of the cooling rate (Ṫ_L_) and growth rate (V_E_), respectively. Experimental relationships (power equations) are displayed to fit the experimental points. The correlation coefficient (R^2^) is a measure of final adjustment applied to the experimental model. The results of the directionally solidified (DS) Sn-9 wt %Zn alloy reported by Ramos et al.^13^ have been included in the graphs for comparative purposes.

The variations of λ_c_ with Ṫ_L_ for the Sn–Zn–Al alloys were characterized by the exponent −0.5. This exponent is similar to that reported by Ramos et al.^13^ for the binary Sn-9 wt %Zn alloy. Other authors have demonstrated similar exponents in tin-based alloys, such as – 0.55 for Sn-9 wt %Zn-2 wt %Cu alloy,^26^ Sn–Bi alloys,^27^ Sn-4 wt %Zn alloy^41^ and Sn–Sb alloys.^28^

It was observed that the 0.5 wt % Al content did not change the microstructural scale of the Sn-9 wt %Zn alloy. However, with the increase in Al content to 2% (in wt %), a small microstructural refinement was observed. This refining effect of Al was reported by Das et al.^18^ and Yavuzer et al.^8^ Al provides greater fluidity, refines grains and improves mechanical resistance.^45^ The presence of Al reduces the size of the tin-rich phase (β-Sn), resulting in a more refined structure and a more homogeneous distribution of the phases.^8^

In Figure 15b, it is observed that a single exponent, −0.5, characterized the variation of λ_n_ with V_E_ for the Sn–Zn–Al alloys, indicating that the Al content did not affect the scale of the eutectic mixture if both ternary alloys are compared. According to Oliveira et al.,^46^ the characterization of the evolution of λ_n_ as a function of V_E_ by a single experimental exponent demonstrates that the interphase spacing directly depends on the heat transfer condition. Therefore, microstructural evolution is influenced by thermal parameters, especially by the growth rate (V). Comparing with the Sn-9 wt %Zn alloy,^13^ it is observed that the Al additions refined the eutectic arrangement, with regard to the needlelike morphology.

### 3.3 Macrosegregation and XRD Analyses

**Figure 16:**
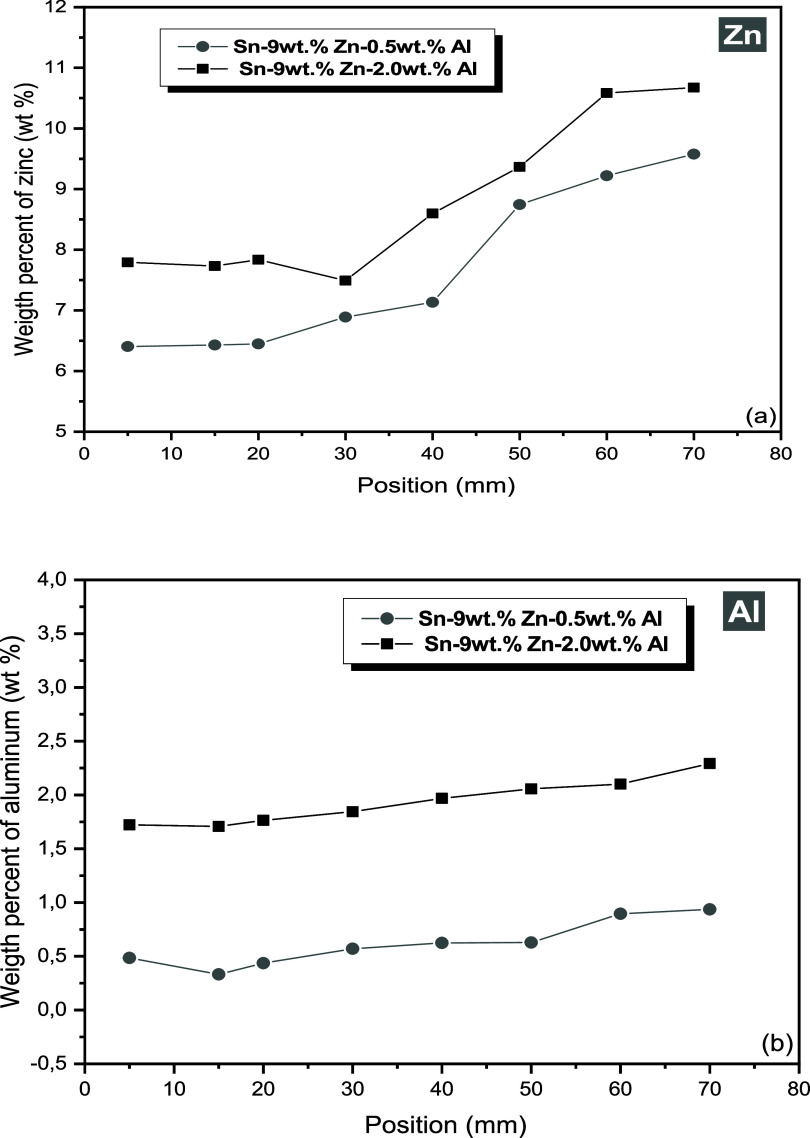
Experimental macrosegregation profiles for (a) zinc and (b) aluminum throughout Sn–Zn–Al castings.

The experimental macrosegregation profiles of Zn and Al along the Sn–Zn–Al alloys are presented in Figure 16. It is noted that the Zn content in the initial positions was lower than the nominal composition, reaching values 9.62 and 10.67%, respectively, for the Sn-9 wt %Zn-0.5 wt %Al and Sn-9 wt %Zn-2.0 wt %Al alloys, at the position 70 mm from the metal/mold interface. This behavior for Zn indicates normal-type macrosegregation profile According to Santos et al.,^47^ the differences in density between Zn (7.14 g/cm^3^) and Sn (7.31 g/cm^3^) result in a Zn profile that tends to increase from the metal/mold interface to the top, considering castings produced by directional solidification in a transient heat-flow regime.

Similarly, it is observed that the Al contents are slightly lower than the nominal compositions in the initial positions of the DS castings, gradually increasing to values of 0.94 and 2.29% for the position at 70 mm from the metal/mold interface, as shown in Figure 15b. Such profiles are characterized as normal type. As reported by Kurz and Fisher,^33^ generally this increase in Al concentration in the last positions can be attributed to the variation in cooling rates throughout the castings. Diffusion at the solid/liquid solidification front is favored for lower values of cooling rates and growth rates. There are few studies in the literature on the solidification behavior of Sn–Zn–Al alloys. One of these works was reported by Oliveira et al.^48^ for the Sn-0.5 wt %Al alloy solidified nonequilibrium on different substrates (nickel and copper). The authors reported that the Al concentration remained essentially close to the nominal value, demonstrating the absence of macrosegregation along the length of the castings.

**Figure 17:**
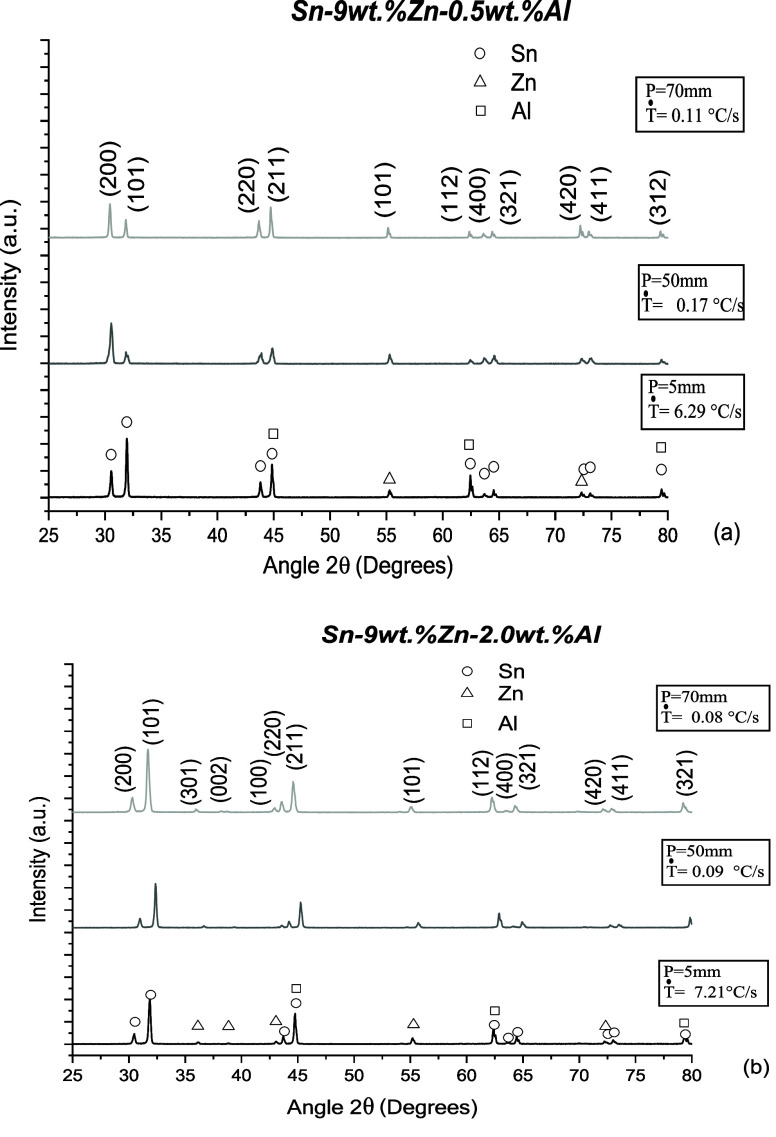
X-ray diffraction patterns for different positions along the (a) Sn-9 wt %Zn-0.5 wt %Al and (b) Sn-9 wt %Zn-2.0 wt %Al castings.

Figure 17 shows the X-ray diffractograms for the Sn–Zn–Al alloys at three distinct and significant positions (5 mm, 50 mm and 70 mm) from the metal/mold interface. The symbols represent the identified phases.

It is noted that the identified peaks are associated with in tin-rich (β-Sn)), zinc-rich (α-Zn) and aluminum-rich (α-Al) phases. These peaks are in accordance with what was predicted by thermodynamic simulation via the CALPHAD method. The crystal planes (200), (101), (220), (211), (112), (400), (321), (420), (411) and (312) confirm the formation of the tetragonal body centered (TBC) crystal structure of the β-Sn phase, while the (301), (002), (100), (101), (420) and (211), (112) and (312) planes attested to the formation of the compact hexagonal (CH) and the face-centered cubic (FCC) crystalline structures for the α-Zn and α-Al phases, respectively.

It is observed that there was no trend in the intensities of the peaks of the Sn-rich phase with the variation of the cooling rate (Ṫ). In contrast, it is noted that the intensities of the Zn-rich phase peaks increase slightly with decreasing Ṫ. This is related to the type-normal Zn macrosegregation profile in the Sn–Zn–Al castings. These results are consistent with the results reported by Eid et al.,^49^ that studied the Sn-9 wt %Zn-0.5 wt %Al alloy and verified the presence of peaks associated with the planes (200), (101), (211) and (321) and 2θ of 31.2°, 33.6°, 45.3° and 64.8° values, respectively, confirming the formation of the tetragonal body centered (TBC) crystal structure of the β-Sn phase. On the other hand, no Al element peak was observed by El Basaty et al.,^19^ which disagrees with the present findings.

### 3.4 Tensile Properties-Microstructure Interrelations

**Figure 18:**
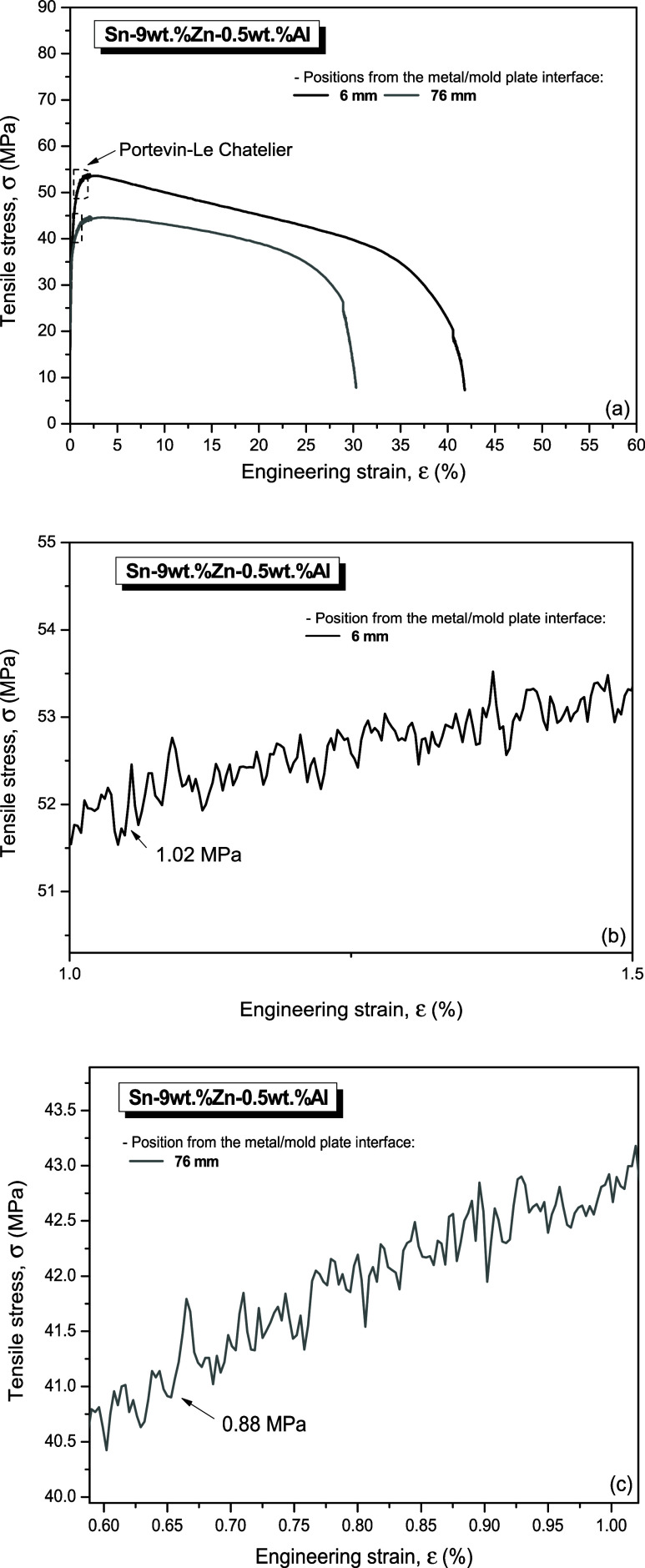
(a) Stress x strain curves referring to positions 6 mm and 76 mm from the metal/mold interface for the Sn-9 wt %Zn-0.5 wt %Al alloy; and (b, c) details of the serrations indicating the presence of the PLC effect.

**Figure 19:**
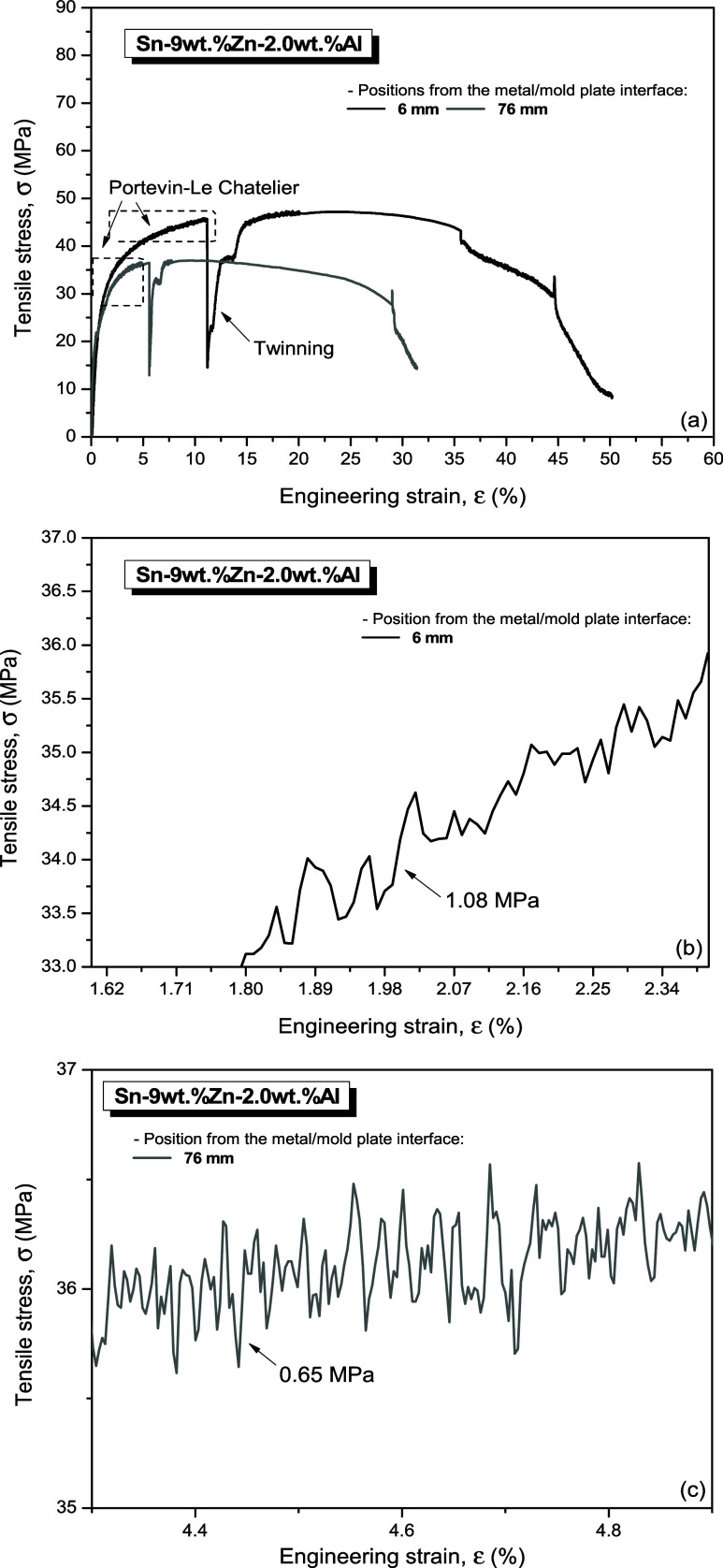
(a) Stress x strain curves referring to positions 6 mm and 76 mm from the metal/mold interface for the Sn-9 wt %Zn-2.0 wt %Al alloy; and (b, c) details of the serrations indicating the presence of the PLC effect.

Figures 18 and 19 show some typical stress x strain curves of the Sn-9 wt %Zn-0.5 wt %Al and Sn-9 wt %Zn-2.0 wt %Al samples, respectively, corresponding to extreme examined positions at 6 mm (black, refined microstructure) and at 76 mm (gray line, coarse microstructure) from the metal/mold interface. Each curve represents a different position in relation to the metal/mold interface, which is associated with a different microstructural scale (λ_C_) and its respective thermal variables (Ṫ_L_ and V_L_) of solidification. For comparative purposes, data from the Sn-9 wt % Zn alloy reported by Ramos et al.^13^ were also inserted.

In general, it is observed that the highest values for mechanical resistance are associated with positions closer to the metal/mold interface, in this case, 6 mm. This behavior is attributed to the more refined cellular and eutectic arrangements at this position, compared to the sample having coarser microstructural arrangement (i.e., 76 mm). The hardening mechanism in Sn–Zn–Al alloys is grain refining,^50^ which says that the smaller the microstructural scale, the greater the mechanical resistance of the material, as the refinement promotes a higher fraction of grain boundaries, which are barriers to the sliding of dislocations in the alloy slip systems.

Moreover, observing Figures 18b,c and 19b,c, the Portevin-Le Chatelier (PLC) effect could be observed. This manifests itself as an unstable plastic flow during tensile tests on metallic materials under certain strain rate and temperature regimes. Plastic deformation becomes localized in the form of bands that move along a sample gauge in various ways as the effect occurs.^51^

According to Li et al.,^52^ the origin of the PLC effect is based on a model called dynamic strain aging (Dynamic Strain Aging-DSA), which is defined as the interaction between moving dislocations and Solute atoms in diffusion. Mobile dislocations act as carriers of plastic deformation according to this mechanism, and move unsteadily between obstacles formed by other defects (substitutional and interstitial) in the material.

The PLC effect can occur in aluminum, copper, zirconium and steel alloys. This mechanism affects most alloy properties. It can increase ultimate tensile strength, decreases ductility of metals with a corresponding decrease in elongation and fracture toughness. Alloys under load become susceptible to unprecedented service failures due to increased embrittlement and reduced fracture toughness resulting from the PLC effect.^51^

It is verified for the refined and coarse samples that the average stress amplitudes of the serrations are 1.02 MPa (Figure 18b) and 0.88 MPa (Figure 18c), respectively, in the Sn-9 wt %Zn-0.5 wt %Al alloy. While average amplitudes in the serrations of 1.08 MPa (Figure 19b) and 0.65 MPa (Figure 19c), respectively, were observed in the Sn-9 wt %Zn-2.0 wt %Al alloy samples. In both cases, the average serration amplitude decreased as the microstructural spacing increased. The same was observed by Schön et al.^28^

Schön et al.^28^ showed stress x strain curves obtained for the Sn-2 wt %Sb alloy associated with different cooling rates 0.9 °C/s, 0.1 °C/s and 0.03 °C/s. The authors found that smaller and more widely spaced PLC bands prevailed in samples with cellular microstructure, as the solute was more available to repeatedly block and release dislocation movements during deformation in the plastic regime.

In Figure 19, related to alloy containing higher Al, it is observed that a transition in the deformation mechanism occurred, going from sliding of crystalline planes to twinning. According to Reed-Hill et al.,^53^ the formation of twins results in sudden increase in the tensile strain, giving the stress–strain curve a sawtooth appearance in the twinning region. When plane sliding is difficult, plastic deformation can occur through twinning. This deformation can occur in materials with a crystalline structure that presents few slip systems or during plastic deformation at low temperatures or high deformation speeds.^50^

The occurrence of plastic deformation through twinning is influenced by stacking defect energy (SDE). The decrease in SDE results in an increasing decrease in the mobility of dislocations, disfavoring deformation by sliding and favoring twinning.^50,53^ The presence of more solute atoms in the material modifies the stacking defect energy. According to Reed-Hill et al.,^53^ SDE decreases with increasing concentration of a solute in solid solution, so that twinning becomes increasingly significant the higher the solute concentration, as proved here through the features observed for the tested samples of the alloy with higher Al content. In metals with a body-centered cubic (BCC) crystal structure, the twinning plane and direction are (111) and [112̅], respectively. These metals have twinning deformation that is highly sensitive to the SDE value.^50^

Thus, it is possible to verify that the increase in the Al content in solid solution in the Sn matrix favored the activation of the twinning deformation mechanism, promoting unfavorable crystalline orientations for the sliding mechanism of crystalline planes in the Sn-9 wt %Zn-2.0 wt %Al alloy.

**Figure 20:**
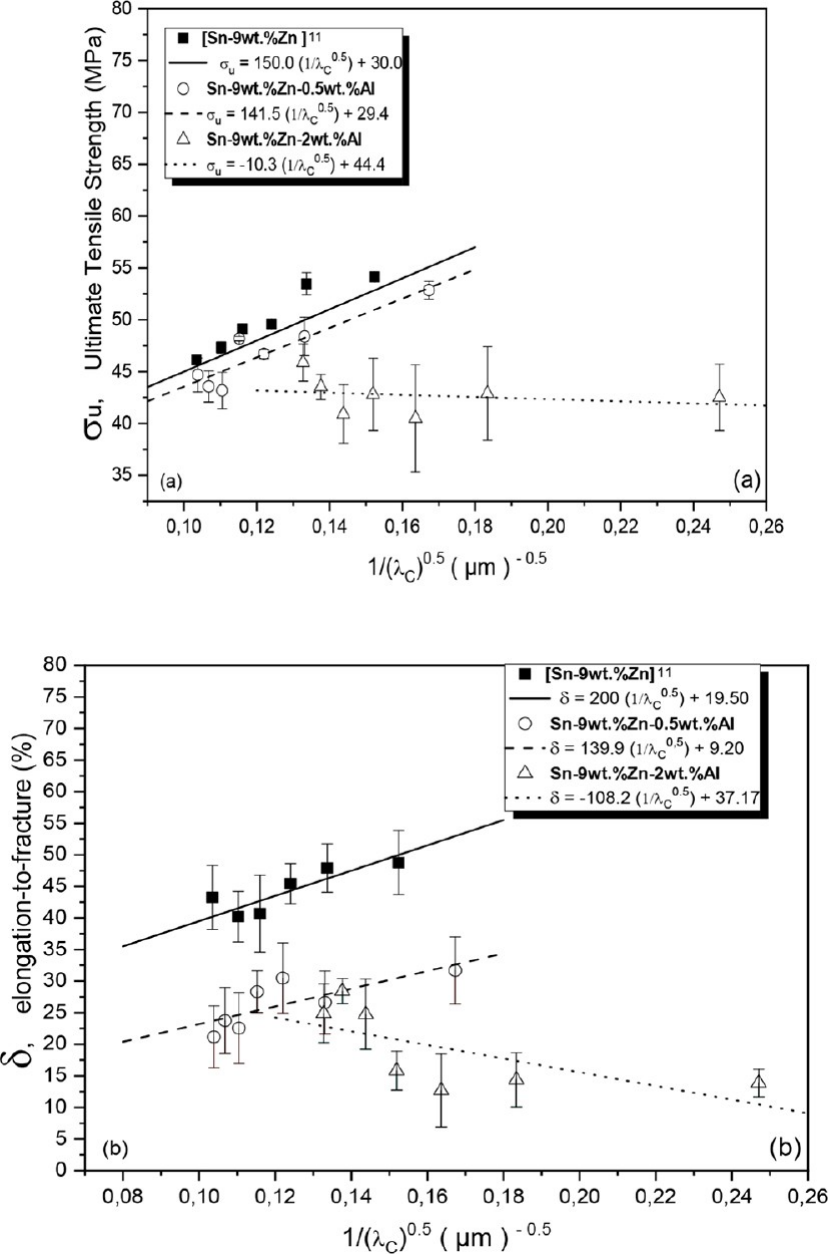
(a) Ultimate tensile strength (σ_u_) and (b) elongation-to-fracture (δ) as a function of the inverse of the cell eutectic spacing (λ_c_) for the Sn–Zn–Al alloy castings.

Figure 20 displays the experimental correlations for the ultimate tensile strength (σ_u_) and elongation-to-fracture (δ) as a function of the cell eutectic spacing (λ_c_) for the Sn–Zn–Al alloys. Each point represents an average of three measurements, with its respective standard deviation. All experimental data were fitted using Hall-Petch type correlations. It is noted that the σ_u_ values are lower than those of the Sn-9 wt %Zn alloy,^13^ especially for more refined microstructures, λ_c_^1/2^ ∼ 0.16.

Considering the samples taken from positions at 6 mm and at 76 mm from the metal/mold interface for each alloy studied, mechanical strength values of 54.1 MPa (λ_c_ = 22.70 μm) and 47.3 MPa (λ_c_ = 82.30 μm), 53.95 MPa (λ_c_ = 37.90 μm) and 44.6 MPa (λ_c_ = 87.70 μm), and 45.8 MPa (λ_c_ = 17.70 μm) and 36.4 MPa (λ_c_ = 52.80 μm), were observed for the Sn-9 wt %Zn,^13^ Sn-9 wt %Zn-0.5 wt %Al and Sn-9 wt %Zn-2.0 wt %Al alloys, respectively. Compared to the Sn-9 wt %Zn alloy, it can be seen that the addition of 0.5 wt %Al in the Sn-9 wt %Zn alloy slightly reduced the σ_u_ values by 0.33% and 5.72%, respectively. The increase in Al content from 0.5% to 2.0% (in wt %) caused a reduction in the σ_u_ values by 15.37% and 23.07%, respectively, highlighting the negative influence of the increase in Al content.

Considering the Sn-9 wt %Zn-0.5 wt %Al alloy, it is noted that the lower λ_c_, the higher the ultimate tensile strength values, indicating that the alloy was mechanically hardened by the grain refining mechanism. However, for the Sn-9 wt %Zn-2.0 wt %Al alloy, there was no variation in σ_u_ values, indicating that the cellular-length scale and microstructural arrangement did not influence the mechanical strength of this alloy.

DAS et al.^18^ analyzed the influence of the addition of Al (0.5 and 0.9 in wt %) on the mechanical properties of Sn-9 wt %Zn-xAl alloys. The ultimate tensile strength values (σ_u_) of the Sn-9 wt %Zn, Sn-9 wt %Zn-0.5 wt %Al and Sn-9 wt %Zn-0.5 wt %Cu alloys were 52, 63, and 48 MPa, respectively. The Sn-9%Zn-0.5 wt %Al alloy had the highest σ_u_ and on the other hand, the Sn-9 wt %Zn alloy exhibited the highest elongation-to-fracture. Yavuzer et al.^8^ observed that the σ_u_ values were 38 MPa (Sn-9 wt %Zn), 35 MPa (Sn-9 wt %Zn-0.7 wt %Cu), 37 MPa (Sn-9 wt %Zn- 0.9 wt %Cu), 45 MPa (Sn-9 wt %Zn-0.7 wt %Al) and 46 MPa (Sn-9 wt %Zn-0.9 wt %Al).

In Figure 20b, it can be seen that the levels of elongation-to-fracture (δ) of the alloys containing Al are lower than those of the Sn-9 wt %Zn alloy, indicating that the such addition did not promote improvements in ductility in the present solidification conditions. It seems that the Al present in eutectic cells did not favor the slipping of dislocations either through the deformation mechanism due to sliding of crystalline planes, nor through the twinning mechanism. Das et al.^18^ observed a 32% reduction in the elongation of the Sn-9 wt %Zn-0.5 wt % Al alloy compared to the Sn-9 wt %Zn alloy. The authors reported that this reduction is associated with an Al-rich phase that precipitated in the microstructure, blocking dislocation slip. Similar results were reported by Yavuzer et al.^8^

In the Sn-9 wt %Zn-0.5 wt %Al alloy, a reduction in cell eutectic spacing (λ_c_) caused an increase in elongation-to-fracture (δ). However, in the Sn-9 wt %Zn-2.0 wt %Al alloy, the highest δ values are associated with coarser cellular arrangements.

**Figure 21:**
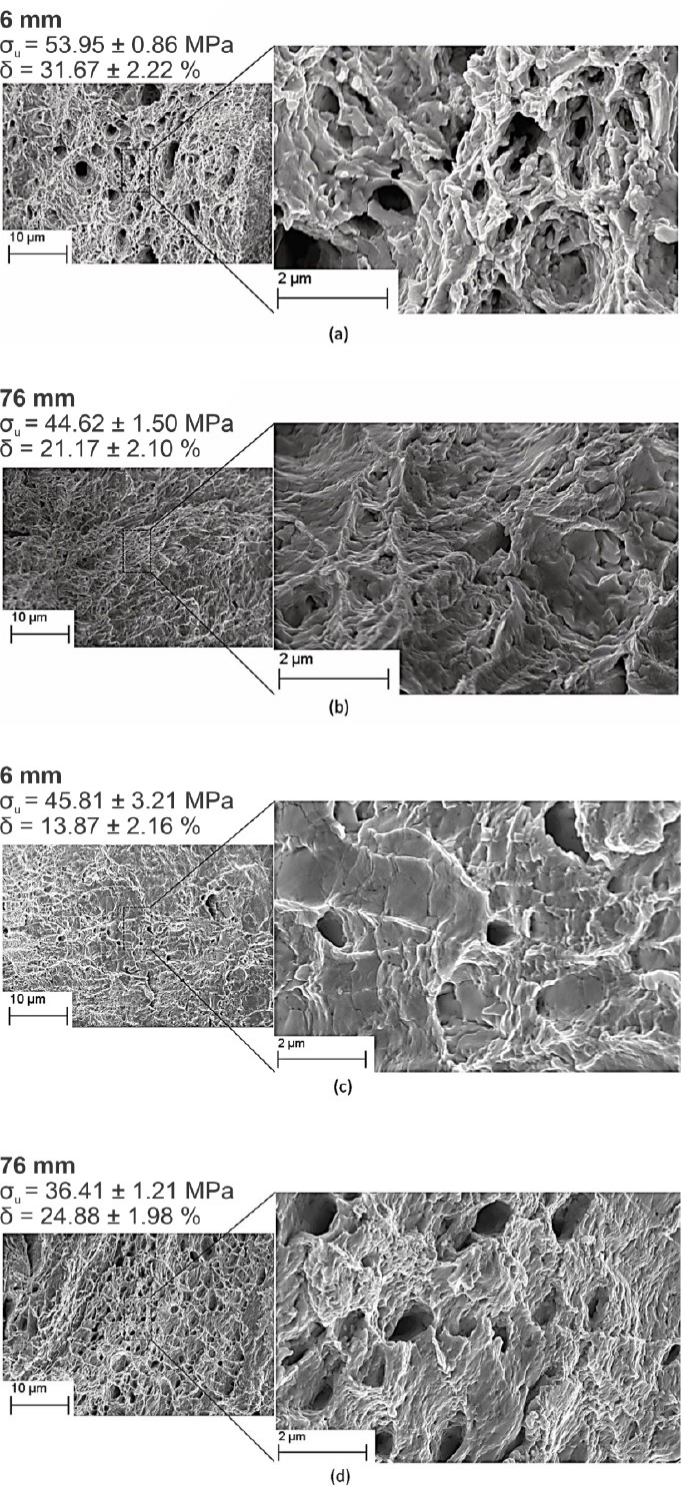
SEM images of the fracture surfaces in the ternary (a,b) Sn-9 wt %Zn-0.5 wt %Al alloy and (c,d) Sn-9 wt %Zn-2.0 wt %Al for positions at 6 mm and at 76 mm from the metal/mold interface.

Figure 21 shows the fracture surfaces associated with the positions of at 6 mm and at 76 mm from the metal/mold interface in the Sn–Zn–Al alloys. In addition, the ultimate Tensile Strength (σ_u_) and elongation-to-fracture (δ) values were inserted for representing each case.

A ductile fracture mode is noted for both Sn–Zn–Al alloys, as evidenced by the presence of dimples. Ramos et al.^13^ reported that the fracture surfaces for the Sn-9 wt %Zn alloy were characterized by regions of ductile fracture. An extensive fraction of alveoli was observed, resulting in high levels of plastic deformation. In this sense, it appears that the addition of Al did not promote changes in the fracture mode of the Sn-9 wt %Zn alloy, despite causing a reduction in the elongation-to-fracture.

## 4 Conclusions

The following conclusions can be drawn from the present experimental work:

- It was noted that Al did not alter the macrosegregation profile of Zn, which continued as normal-type throughout the Sn–Zn–Al castings.
- It was noted that the increase in Al content generated an increase in the *liquidus* temperature (T_L_) and a reduction in the eutectic temperature (T_E_), increasing the alloy solidification interval.
- A eutectic cellular microstructure composed of β-Sn, α-Zn and α-Al phases has been observed for Sn–Zn–Al alloys. The morphological transitions of the α-Zn phase were shown to be dependent on the cooling rate (Ṫ) and the Zn and Al contents.
- The addition of 2 wt %Al content promoted a significant refinement in the eutectic cellular arrangement, when compared to the Sn-9 wt %Zn alloy. Al additions refined the scale of the eutectic mixture (interphase spacing-λ_n_), without causing changes due to increasing Al content considering the Sn−Zn−Al alloys.
- The following experimental power functions relating the cell spacing (λ_c_: μm) to the cooling rate, λ_c_= 35 (Ṫ) ^–^°.^5^ and λ_c_= 24 (Ṫ) ^–^°.^5^, were shown to represent the eutectic cellular growth for both castings, i.e., Sn-9 wt %Zn-0.5 wt %Al alloy and Sn-9 wt %Zn-2.0 wt %Al, respectively.
- The PLC effect was observed in the stress x strain curves of the Sn–Zn–Al alloys, but especially for the modified alloy with higher Al content. It was observed that Al additions favored the activation of the twinning deformation mechanism in the Sn–Zn–Al alloys.
- Al additions promoted reductions in both the ultimate tensile strength (σ_u_) and the elongation-to-fracture (δ). Despite the reduction in ductility of the Sn–Zn–Al alloys, Al did not change the fracture mode of the Sn-9 wt %Zn alloy regardless of the content used, remaining ductile in character with the prevalence of dimples.

## References

[ref1] Ahmido A.; Sabbar A.; Zouihri H.; Dakhsi K.; Guedira F.; Serghini-Idrissi M. Effect of bismuth and silver on the corrosion behavior of Sn–9Zn alloy in NaCl 3 wt.% solution. Mater. Sci. Eng. 2011, 176, 1032–1036. 10.1016/j.mseb.2011.05.034.

[ref2] Chen W.; Xue S.; Wang H.; Hu Y.; Wang J. Effects Of Rare Earth Ce On Properties Of Sn–9zn Lead-Free Solder. Journal Of Materials Science. Mater. Electron. 2010, 21, 719–725. 10.1007/S10854-009-9984-2.

[ref3] Directive E. Restriction of the use of certain hazardous substances in electrical and electronic equipment (RoHS). Off. J. Eur. Commun., 2013, 46, 19, 23.

[ref4] Label, P. Ohs Compliance Engineer R, Directive 2002/95/EC Of The European Parliament And Of The Council Of 27 January 2003 On The Restriction Of The Use Of Certain Hazardous Substances In Electrical And Electronic Equipment Artesyn Technologies 2005 48

[ref5] Chen X.; Hu A.; Li M.; Mao D. Study on the properties of Sn–9Zn–xCr leadfree solder. J. Alloys Compd. 2008, 460, 478–484. 10.1016/j.jallcom.2007.05.087.

[ref6] Liou W.-K.; Yen Y.-W.; Chen K.-D. Interfacial reactions between Sn-9Zn^+^Cu leadfree solders and the Au substrate. J. Alloy. Comp. 2009, 479, 225–229. 10.1016/j.jallcom.2008.12.141.

[ref7] Mehreen S. U.; Nogita K.; Mcdonald S. D.; Yasuda H.; StJohn D. H. Peritectic phase formation kinetics of directionally solidifying Sn-Cu alloys within a broad growth rate regime. Acta Mater. 2021, 220, 117295 10.1016/j.actamat.2021.117295.

[ref8] Yavuzer B.; Özyurek D.; Tunçay T. Microstructure and mechanical properties of Sn-9Zn-xAl and Sn-9Zn-xCu lead-free solder alloys. Materials Science-Poland 2020, 38, 34–40. 10.2478/msp-2020-0025.

[ref9] Affendy M. G.; Mohamad A. A. Effects Of Crosshead Speeds On Solder Strength Of Cu/Sn– 9Zn/Cu Lap Joints. J. King. Saud. Univ. Eng. Sci. 2015, 27, 225–231. 10.1016/j.jksues.2013.09.003.

[ref10] Nazeri M. F.; Mohamad A. A. Corrosion resistance of ternary Sn-9Zn-xIn solder joint in alkaline solution. J. Alloys Compd. 2016, 661, 516–525. 10.1016/j.jallcom.2015.11.184.

[ref11] Dybeł A.; Pstrú J. New solder based on the Sn-Zn eutectic with addition of Ag, Al, and Li. J. Mater. Eng. Perform. 2023, 32 (13), 5710–5722. 10.1007/s11665-023-08103-0.

[ref12] González-Parra R.; Novelo-Peralta O.; Lara-Rodríguez G.; Figueroa I.; Barba A.; Hernandez M. Influence of alloying elements on microstructure, mechanical properties and corrosion behaviour of hypoeutectic Sn-6.5 wt% Zn-0.5 wt% X (X= Ag, Al, Cu) lead-free solders. J. Mater. Sci.: Mater. Electron. 2024, 35 (22), 1539.

[ref13] Ramos L. S.; Reyes R. V.; Gomes L. F.; Garcia A.; Spinelli J. E.; Silva B. L. The role of eutectic colonies in the tensile properties of a Sn–Zn eutectic solder alloy. Mater. Sci. Eng., A 2020, 776, 138959 10.1016/j.msea.2020.138959.

[ref14] Zhang L.; Xue S. B.; Gao L.-I.; Sheng Z. Development Of Sn–Zn Lead-free Solders Bearing Alloying Elements. J. Mater. Sci.: Mater. Electron. 2010, 21, 1–15. 10.1007/s10854-009-0014-1.

[ref15] Kitajima M.; Shono T. Development of Sn-Zn-Al lead-free solder alloys. Fujitsu Sci. Technical. J. 2005, 41, 225–235.

[ref16] Lin K. L.; Liu T. P. High-temperature oxidation of a Sn-Zn-Al solder. Oxid. Met. 1998, 50, 255–267. 10.1023/A:1018840405283.

[ref17] Zhang Z.; Liu Y.; Ma T.; Zhang S.; Yang X.; Shao W.; Yang J.; Chen S.; Ye Z.; Wang W.; Yang J. Influence of Pt addition on corrosion resistance of Sn-9Zn-0.02 Al-xPt solder alloys. Corros. Sci. 2024, 240, 112430 10.1016/j.corsci.2024.112430.

[ref18] Das S. K.; Sharif A.; Chan Y.; Wong N. B.; Yung W. K. C. Influence of small amount of Al and Cu on the microstructure, microhardness and tensile properties of Sn–9Zn binary eutectic solder alloy. J. Alloys Compd. 2009, 482, 167–172. 10.1016/j.jallcom.2009.03.017.

[ref19] El Basaty A.; Deghady A.; Eid E. Influence of small addition of antimony (Sb) on thermal behavior, microstructural and tensile properties of Sn-9.0 Zn-0.5 Al Pb-free solder alloy. Mater. Sci. Eng., A 2017, 701, 245–253. 10.1016/j.msea.2017.06.092.

[ref20] Ma X.; Xiu Z.; Xu Y.; Yan J. Ultrasonic-assisted soldering of sapphire through metallic transition layers of Al and Zn using Sn-xZn-2Al solder alloys. Ceram. Int. 2023, 49 (2), 2451–2460. 10.1016/j.ceramint.2022.09.218.

[ref21] Shen J.; Liu Y.; Han Y.; Gao H.; Wei C.; Yang Y. Effects of cooling rates on microstructure and microhardness of lead-free Sn-3.5% Ag solders, T. Nonferr. Metal. Soc. 2006, 16, 59–64. 10.1016/S1003-6326(06)60011-3.

[ref22] Moura I. T. L.; Silva C. L. M.; Cheung N.; Goulart P. R.; Garcia A.; Spinelli J. E. Cellular to dendritic transition during transient solidification of a eutectic Sn 0.7 wt% Cu solder alloy. Mater. Chem. Phys. 2012, 132, 203–209. 10.1016/j.matchemphys.2011.11.033.

[ref23] Silva B. L.; Cheung N.; Garcia A.; Spinelli J. E. Thermal parameters, microstructure, andmechanical properties of directionally solidified Sn-0.7 wt% Cu solder alloys containing 0 ppm to 1000 ppm Ni. J. Electron. Mater. 2013, 42, 179–191. 10.1007/s11664-012-2263-7.

[ref24] Hu X.; Li Y.; Liu Y.; Min Z. Developments of high strength Bi-containing Sn0.7Cu lead-free solder alloys prepared by directional solidification. J. Alloys Compd. 2015, 65, 241–250. 10.1016/j.jallcom.2014.10.205.

[ref25] Santos W. L. R.; Silva B. L.; Bertelli F.; Spinelli J. E.; Cheung N.; Garcia A. An alternative termal approach to evaluate the wettability of solder alloys. Appl. Therm. Eng. 2016, 107, 431–440. 10.1016/j.applthermaleng.2016.06.177.

[ref26] Silva B. L.; Reyes R. V.; Garcia A.; Spinelli J. E. Dendritic Growth, Eutectic Features and Their Effects on Hardness of a Ternary Sn–Zn–Cu Solder Alloy. Acta Metall. Sin. 2017, 30, 528–540. 10.1007/s40195-017-0572-9.

[ref27] Silva B. S.; Garcia A.; Spinelli J. E. Complex eutectic growth and Bi precipitation in ternary Sn Bi-Cu and Sn-Bi-Ag alloys. J. Alloys Compd. 2017, 691, 600–605. 10.1016/j.jallcom.2016.09.003.

[ref28] Schon A. F.; Reyes R. V.; Spinelli J. E.; Garcia A.; Silva B. L. Assessing microstructure and mechanical behavior changes in a Sn-Sb solder alloy induced by cooling rate. J. Alloys Compd. 2019, 809, 151780 10.1016/j.jallcom.2019.151780.

[ref29] Silva B. L.; Spinelli J. E. Correlations Of Microstructure And Mechanical Properties Of The Ternary Sn-9wt% Zn-2wt% Cu Solder Alloy. Mater. Res. 2018, 21, 20170877 10.1590/1980-5373-MR-2017-0877.

[ref30] Sobral B. S.; Vieira P. S.; Lima T. S.; Spinelli J. E.; Cheung N.; Garcia A.; Silva B. L. Effects Of Zn Addition On Dendritic/Cellular Growth, Phase Formation, And Hardness Of a Sn-3.5wt% Ag Solder Alloy. Adv. Eng. Mater. 2023, 25, 2201270 10.1002/adem.202201270.

[ref31] Mccartney D.; Hunt J. Measurements of cell and primary dendrite arm spacings in directionally solidified aluminium alloys. Acta Metall. 1981, 29, 1851–1863. 10.1016/0001-6160(81)90111-5.

[ref32] Astm, Standard test methods for tension testing of metallic materials. In Annual book of ASTM standards; ASTM, 2001.

[ref33] Kurz W.; Fisher D. J. Fundamentals of solidification; Trans Tech Publications: Zurich, 1998.

[ref34] Dias M.; Costa T. A.; Silva B. L.; Spinelli J. E.; Cheung N.; Garcia A. A comparative analysis of microstructuralfeatures, tensile properties and wettability of hypoperitectic and peritectic SnSb solder alloys. Microelectron. Reliab. 2018, 81, 150–158. 10.1016/j.microrel.2017.12.029.

[ref35] Smetana B.; Zlá S.; Kroupa A.; Žaludová M.; Drápala J.; Burkovič R.; Petlák D. Phase transition temperatures of Sn–Zn–Al system and their comparison with calculated phase diagrams. J. Therm. Anal. Calorim. 2012, 110 (1), 369–378. 10.1007/s10973-012-2318-2.

[ref36] Lu B.; Li Y.; Yu W.; Wang H.; Xu Z.; Wang Z.; Xu G. Strength and ductility enhancement of twin-roll cast Al-Zn-Mg-Cu alloys with high solidification intervals through a synergistic segregation-controlling strategy. J. Mater. Sci. Technol. 2023, 142, 225–239. 10.1016/j.jmst.2022.09.033.

[ref37] Flemings M. C. Our Understanding Of Macrosegregation: Past And Present. ISIJ. Intern. 2000, 40, 833–884. 10.2355/isijinternational.40.833.

[ref38] Hulka K.; Gray J. M. High temperature processing of line-pipe steels International Symposium Niobium Niobium 2001 587–612

[ref39] Gouveia G. L.; Gomes L. F.; Cheung N.; Garcia A.; Spinelli J. E. Mechanical Properties, Microstructural Features, and Correlations with Solidification Rates of Al–Cu–Si Ultrafine Eutectic Alloys. Adv. Eng. Mater 2021, 23 (4), 2001177 10.1002/adem.202001177.

[ref40] Gomes L. F.; Silva B.; Silva Júnior P. S.; Garcia A.; Spinelli J. E. Ag-containing aluminum- silicon alloys as an alternative for as-cast components of electric vehicles. Mater. Res. Express 2021, 8 (1), 016527 10.1088/2053-1591/abdabe.

[ref41] Garcia L. R.; Osório W. R.; Wislei R.; Peixoto L. C.; Garcia A. Wetting Behavior and Mechanical Properties of Sn-Zn and Sn-Pb Solder Alloys. J. Electron. Mater. 2009, 38 (11), 2405–2414. 10.1007/s11664-009-0888-y.

[ref42] Garcia L. R.; Osório W. R.; Peixoto L. C.; Garcia A. Mechanical Properties Of Sn–zn Lead-Free Solder Alloys Based On The Microstructure Array. Mater. Charact. 2010, 61, 212–220. 10.1016/j.matchar.2009.11.012.

[ref43] Song J.-M.; Wu Z.-M. Variable eutectic temperature caused by inhomogeneous solute distribution in Sn–Zn system. Scr. Mater. 2006, 54 (8), 1479–1483. 10.1016/j.scriptamat.2005.12.056.

[ref44] Moser Z.; Dutkiewicz J.; Gasior W.; Salawa J. The Sn– Zn (Tin-Zinc) system. J. Phase Equil. 1985, 6 (4), 330–334. 10.1007/BF02880511.

[ref45] Prasad B. K. Effect Of Microstructure On The Sliding Wear Performance Of a Zn-Al-Ni Alloy. Wear 2000, 240, 100–112. 10.1016/S0043-1648(00)00369-0.

[ref46] Oliveira R.; Cruz C. B.; Barros A.; Bertelli F.; Spinelli J. E.; Garcia A.; Cheung N. Thermal conductance at Sn-0.5 mass% Al solder alloy/substrate interface as a factor for tailoring cellular/dendritic growth. J. Therm. Anal. Calorim. 2022, 147 (8), 4945–4958. 10.1007/s10973-021-10755-w.

[ref47] Santos W. L. R.; Cruz C. B.; Spinelli J. E.; Cheung N.; Garcia A. Tailoring Microstructure, Tensile Properties And Fracture Process Via Transient Directional Solidification Of Zn-Sn Alloys. Mater. Scie. Eng.: A 2018, 712, 127–132. 10.1016/j.msea.2017.11.039.

[ref48] Oliveira R.; et al. Thermal conductance at Sn-0.5 mass% Al solder alloy/substrate interface as a factor for tailoring cellular/dendritic growth. J. Therm. Anal. Calorim. 2022, 147, 4945–4958. 10.1007/s10973-021-10755-w.

[ref49] Eid E.; Ramadan M. A.; El Basaty A. Enhancing the creep resistance of Sn9.0 Zn-0.5 Al lead- free solder alloy by small additions of Sb element. Engineering 2018, 10, 21 10.4236/eng.2018.101003.

[ref50] Meyers M. A.; Chawla K. K. Mechanical Metallurgy: principles and Application, Prentice-Hall, Inc., 1982.

[ref51] Yilmaz A. *Portevin–Le Chatelier Effect: a Review Of Experimental Findings*. Sci. Technol. Adv. Mater. 2011, 12, 063001 10.1088/1468-6996/12/6/063001. 27877450 PMC5090665

[ref52] Li C.; Teng J.; Yang B.; Ye X.; Huang L.; Liu Y.; Li Y. Portevin-Le Châtelier Effect in a Powder Metallurgy Co-Ni-Based Superalloy. Materials 2022, 15 (8), 2796 10.3390/ma15082796. 35454489 PMC9028865

[ref53] Reed-Hill R. E.; Abbaschian R. Physical metallurgy principles; Van Nostrand: New York, 1973.

